# *Hemgn* Protects Hematopoietic Stem and Progenitor Cells Against Transplantation Stress Through Negatively Regulating IFN‐*γ* Signaling

**DOI:** 10.1002/advs.202103838

**Published:** 2021-12-19

**Authors:** Ke Zhao, Jin‐Fang Liu, Ya‐Xin Zhu, Xiao‐Ming Dong, Rong‐Hua Yin, Xian Liu, Hui‐Ying Gao, Feng‐Jun Xiao, Rui Gao, Qi Wang, Yi‐Qun Zhan, Miao Yu, Hui Chen, Hong‐Mei Ning, Cai‐Bo Zhang, Xiao‐Ming Yang, Chang‐Yan Li

**Affiliations:** ^1^ State Key Laboratory of Proteomics Beijing Proteome Research Center National Center for Protein Sciences (Beijing) Beijing Institute of Lifeomics Beijing 102206 China; ^2^ School of Life Sciences Hebei University No. 180 Wusi Dong Road, Lian Chi District Baoding City Hebei Province 071000 China; ^3^ College of Life Sciences Shanxi Normal University No. 199, South Chang'an Road, Yanta District Xi'an 710062 China; ^4^ Department of Experimental Hematology and Biochemistry Beijing Institute of Radiation Medicine Beijing 100850 China; ^5^ An Hui Medical University School of Basic Medical Sciences Hefei 230032 China; ^6^ Department of Hematopoietic Stem Cell Transplantation The Fifth Medical Center of Chinese PLA General Hospital Beijing 100071 China; ^7^ Department of Life Sciences Qilu Normal University No. 2, Wenbo Road, Zhangqiu District Jinan Shandong 250013 China

**Keywords:** engraftment defect, hematopoietic stem/progenitor cells, *Hemgn*, IFN‐*γ* signaling

## Abstract

Hematopoietic stem and progenitor cells (HSPCs) possess the remarkable ability to regenerate the whole blood system in response to ablated stress demands. Delineating the mechanisms that maintain HSPCs during regenerative stresses is increasingly important. Here, it is shown that *Hemgn* is significantly induced by hematopoietic stresses including irradiation and bone marrow transplantation (BMT). *Hemgn* deficiency does not disturb steady‐state hematopoiesis in young mice. *Hemgn* ^−/‐^ HSPCs display defective engraftment activity during BMT with reduced homing and survival and increased apoptosis. Transcriptome profiling analysis reveals that upregulated genes in transplanted *Hemgn* ^−/−^ HSPCs are enriched for gene sets related to interferon gamma (IFN‐*γ*) signaling. *Hemgn* ^−/−^ HSPCs show enhanced responses to IFN‐*γ* treatment and increased aging over time. Blocking IFN‐*γ* signaling in irradiated recipients either pharmacologically or genetically rescues *Hemgn* ^−/‐^ HSPCs engraftment defect. Mechanistical studies reveal that *Hemgn* deficiency sustain nuclear Stat1 tyrosine phosphorylation via suppressing T‐cell protein tyrosine phosphatase TC45 activity. Spermidine, a selective activator of TC45, rescues exacerbated phenotype of HSPCs in IFN‐*γ*‐treated *Hemgn* ^−/−^ mice. Collectively, these results identify that *Hemgn* is a critical regulator for successful engraftment and reconstitution of HSPCs in mice through negatively regulating IFN‐*γ* signaling. Targeted *Hemgn* may be used to improve conditioning regimens and engraftment during HSPCs transplantation.

## Graphical abstract

HEMGN is induced by bone marrow transplantation (BMT) stress which in turn negatively regulates IFN‐*γ* signaling through promoting TC45‐medicated Stat1 dephosphorylation, resulting in successful engraftment and reconstitution of donor HSPCs. This study provides novel insight into how HSPCs combat transplantation stress to replenish a damaged hematopoietic system. Targeted *Hemgn* may be used to improve conditioning regimens and engraftment during BMT.

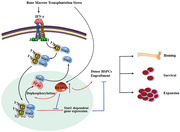

## 1 Introduction

Hematopoietic stem and progenitor cells (HSPCs) possess the remarkable ability to replenish the blood system and maintain hematopoietic homeostasis in response to either physiological or ablated stress demands, such as irradiation, chemotherapy, or hematological diseases.^[^ ^1^, ^2^ ^]^ This ability is exploited routinely in the clinic via HSPCs transplantation (HSPCT).^[^ ^3^ ^]^ Successful HSPCT depends on the engraftment process, in which donor cells are lodged in the bone marrow (BM) medullary cavity, and subsequent retention and proliferation in the BM space leads to reconstitution of the hematopoietic system.^[^ ^4^ ^]^ Ionizing radiation is used to condition patients receiving HSPCT, which induces a cytokine release syndrome.^[^ ^5^ ^]^ In the early stages of engraftment, donor cells operate within a skewed cytokines environment, need to withstand the transplantation stress which includes multiple apoptotic signals and respond to proliferative stimuli, exiting their quiescent phase and undergoing a period of self‐renewal and differentiation to restore hematopoietic homeostasis. Limited responsiveness to inflammatory cytokines is a feature of transplanted HSCs and contributes to successive engraftment during HSPCT.^[^ ^6^ ^]^ However, the underlying regulatory mechanisms remain unclear. A better understanding of the molecular processes that HSPCs employ to withstand the transplantation stress will illuminate novel targets for improving conditioning regimens and engraftment during HSPCT.

Hemogen (*Hemgn*), homologous to human erythroid differentiation‐associated gene (*EDAG*) and rat *RP59*,^[^ ^7^, ^8^ ^]^ is a vertebrate transcriptional regulator that performs important functions in hematopoietic and testicular development and might contribute to neoplasia.^[^ ^9^, ^10^ ^]^ In hematopoietic cells, *Hemgn* is mainly expressed in active hematopoietic sites and downregulated during blood cell differentiation process. *Hemgn* transcription in hematopoietic cells is regulated by GATA1 and HOXB4.^[^ ^11^, ^12^, ^13^ ^]^ Early studies suggest that *Hemgn* is an important regulator for proliferation, differentiation, survival,^[^ ^10^ ^]^ and resistance to chemotherapy of hematopoietic cells.^[^ ^14^ ^]^ *Hemgn* positively regulates erythroid differentiation of human CD34^+^ cells partially by recruiting histone acetyltransferase p300 to acetylate GATA1.^[^ ^15^ ^]^ A transgenic mouse model driven by human CD11a promoter showed that overexpression of human *Hemgn* suppresses the lymphoid lineage development but enhances myeloid development.^[^ ^16^ ^]^ Recently, *Hemgn* is shown to promote rapid entry of human CD34^+^ cells into the cell cycle, and enhance their proliferative potential and repopulating capacity.^[^ ^17^ ^]^ In addition, *Hemgn* also partially recapitulates the function of HOXB4 in promoting mouse myeloid progenitor cells expansion ex vivo.^[^ ^13^ ^]^ Although these data indicate that *Hemgn* might function as a regulator in HSPCs, the role and mechanism of *Hemgn* in HSPCs is poorly understood. Interestingly, *Hemgn* expression is induced by various stresses such as differentiation induction, proliferation stimulation, irradiation, and hypoxia exposure,^[^ ^10^, ^15^, ^18^, ^19^ ^]^ suggesting that *Hemgn* may play a role in regulation HSPCs function under stress conditions. Here, we focus on whether and how *Hemgn* regulates HSPCs function in response to transplantation stress.

## 2 Results

### 2.1 *Hemgn* Is Dispensable for Steady‐State Hematopoiesis

**Figure 1:**
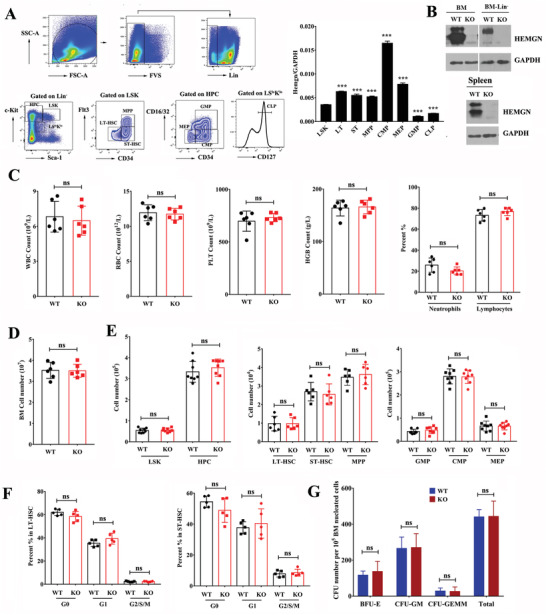
*Hemgn* is dispensable for steady‐state hematopoiesis in young mice. A) *Hemgn* mRNA expression levels in purified murine HSPCs (*n* = 6 mice). The left panel represents the gating strategy for HSPCs and committed progenitors. Data shown are representative of three independent experiments with a total of six mice per group. Each mouse was performed in triplicate. Error bars indicate SEM. B) Validation of *Hemgn* knockout mice by western blotting analysis of HEMGN protein expression in total BM mononuclear cells, BM Lin^−^ cells and spleen from WT and *Hemgn* ^−/−^ (KO) mice. GAPDH was used as internal control. C) *Hemgn* ^−/−^ mice displayed normal cellularity of PB and D) BM (*n* = 6 mice per group). Data are the pool of two independent experiments. Error bars indicate SD. E) The total numbers of the HSPCs and committed progenitors in the BM of *Hemgn* ^−/−^ mice were comparable to those in WT mice (*n* = 8 mice per group). Data are the pool of two independent experiments. Error bars indicate SD. F) Cell cycle analysis of LT‐HSCs and ST‐HSCs in *Hemgn* ^−/−^ and WT BM using DAPI and Ki‐67 staining (*n* = 5 mice per group). Data are the pool of two independent experiments. Error bars indicate SD. G) CFU assays of *Hemgn* ^−/−^ and WT BM cells (*n* = 5 mice per group). Data shown are representative of two independent experiments with a total of five mice per group. Each mouse was performed in triplicate. Error bars indicate SEM. For all graphs, *p < 0.05, **p < 0.01, ***p < 0.001.

We first examined the expression pattern of *Hemgn* in isolated murine HSPCs populations from bone marrow (BM). Like human EDAG, *Hemgn* was expressed at low levels in Lin^−^Sca‐l^+^c‐Kit^+^ (LSK), long‐term HSCs (LT‐HSCs, Lin^−^Sca‐l^+^c‐Kit^+^CD34^−^Flt3^−^), short‐term HSC (ST‐HSCs, Lin^−^Sca‐l^+^c‐Kit^+^CD34^+^Flt3^−^), granulocyte‐monocyte progenitors (GMPs, Lin^−^Sca‐l^−^c‐Kit^+^CD34^+^CD16/32^hi^), and common lymphoid progenitors (CLPs, Lin^−^c‐Kit^low^Sca‐1^low^CD127^+^), with elevated levels observed in committed myeloid progenitors (CMPs, Lin^−^Sca‐l^−^c‐Kit^+^CD34^+^CD16/32^low^) and megakaryocyte‐erythroid progenitors (MEPs, Lin^−^Sca‐l^−^c‐Kit^+^CD34^−^CD16/32^low^) (**Figure** **1**A). To assess the functional role of *Hemgn* in HSPCs, we generated a whole‐body *Hemgn* ^−/−^ mouse model on a C57/BL/6J (CD45.2^+^) background. The mutant allele was confirmed by sequencing the *Hemgn* cDNA (Figure S1, Supporting Information). Western blot analysis using a specific antibody against mouse HEMGN demonstrated that neither intact nor truncated HEMGN proteins were present in *Hemgn* ^−/−^ HSPCs (Figure 1B). *Hemgn* ^−/−^ mice appeared normal and showed no internal anatomical abnormalities (data not shown). In 2‐month‐old animals, *Hemgn* ^−/−^ mice display normal cellularity of peripheral blood (PB) (Figure 1C) and BM (Figure 1D), as well as the constitution of leukocytes in PB. The numbers of immunophenotypically‐defined enriched HSPCs in the BM of *Hemgn* ^−/−^ mice were comparable to those in WT mice (Figure 1E). No significant difference of the cell cycle status in LT‐HSCs and ST‐HSCs was observed between *Hemgn* ^−/−^ and WT mice (Figure 1F; Figure S2, Supporting Information). Functional colony‐forming unit (CFU) assays suggested that the numbers of burst forming unit‐erythroid (BFU‐E), CFU‐granulocyte and macrophage (CFU‐GM), and CFU‐granulocyte, erythrocyte, macrophage, and megakaryocyte (CFU‐GEMM) in BM (Figure 1G) and spleen (SP) (Figure S3, Supporting Information) were similar between *Hemgn* ^−/−^ and WT mice. Serial replating assays suggested that *Hemgn* ^−/−^ and WT HSCs displayed similar self‐renewal capacity (Figure S4, Supporting Information), indicating that the number of functional HSCs in *Hemgn* ^−/−^ mice was comparable to that in WT mice. Overall, these findings suggest that *Hemgn* is dispensable for steady‐state hematopoiesis under homeostatic conditions.

### 2.2 *Hemgn^−/−^* HSPCs Display Severely Defective Engraftment Activity

**Figure 2:**
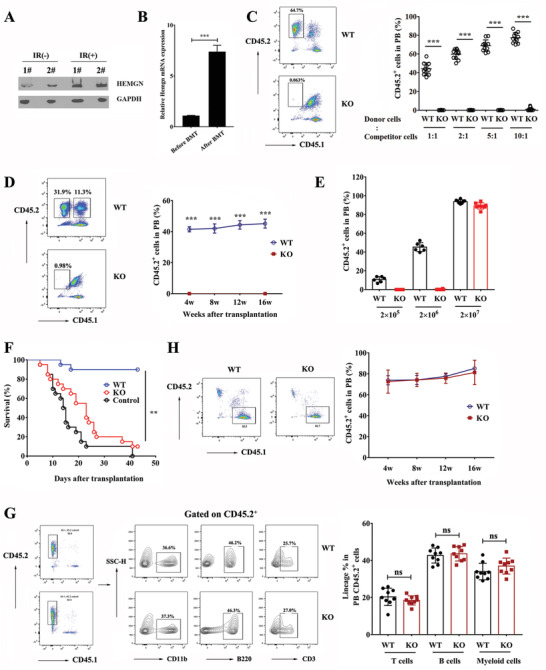
*Hemgn^−/^* ^−^ HSPCs display severely defective engraftment activity. A) Mice were exposed to 4.5Gy TBI for 1h and LSK cells were isolated for measuring HEMGN protein expression. *n* = 2 mice per group. B) *Hemgn* mRNA expression levels in LSK cells before and after transplantation was detected by quantitative RT‐PCR (*n* = 3 mice in Before BMT group, *n* = 6 mice in After BMT group). Each mouse was performed in triplicate. Error bars indicate SEM. C) Whole BM cells from WT and *Hemgn* ^−/−^ mice (CD45.2^+^) were mixed with competitor BM cells (CD45.1^+^CD45.2^+^, 1 × 10^6^) at the indicated ratio and injected into lethally irradiated recipients (CD45.1^+^). Donor chimerism in PB was analyzed at 16 weeks after transplantation (*n* = 10 mice per group). The left panel shows representative flow cytometric plots of mice transplanted with WT or *Hemgn* ^−/−^ BM cells with competitor cells (5:1). Data are the pool of two independent experiments. Error bars indicate SD. D) Competitive transplantation assay with BM LSK cells (1 × 10^4^). PB was analyzed for donor chimerism at the indicated time points (*n* = 6 mice per group). The left panel shows representative flow cytometric plots of mice at 16 weeks after transplantation. Data are the pool of two independent experiments. Error bars indicate SD. E) Lethally irradiated CD45.1 mice were transplanted with indicated numbers of donor BM cells and the donor chimerism in PB was analyzed at 4 weeks after transplantation (*n* = 6–9 mice per group). Data are the pool of two independent experiments. Error bars indicate SD. F) Survival curve of lethally irradiated CD45.1 mice transplanted with 5 × 10^5^ donor BM cells (*n* = 19 mice per group). In control group, PBS was injected into recipients without donor BM cells (*n* = 20 mice). Data are the pool of two independent experiments. G) Lethally irradiated CD45.1 mice were transfected with 2 × 10^7^ WT or *Hemgn* ^−/−^ donor BM cells and at 16 weeks after transplantation, the trilineage differentiation in PB was examined by flow cytometry (*n* = 9 mice per group). The left panel showed representative flow cytometric plots of PB chimerism and trilineage differentiation at 16 weeks after transplantation. Data are the pool of two independent experiments. Error bars indicate SD. H) WT BM cells (5 × 10^6^, CD45.1^+^) were transplanted into WT or *Hemgn* ^−/−^ recipients and the chimerism in PB were analyzed by flow cytometry at the indicated time (*n* = 8 mice per group). The left panel showed representative flow cytometric plots of PB chimerism at 16 weeks after transplantation. Data are the pool of two independent experiments. Error bars indicate SD. For all graphs, **p < 0.01, ***p < 0.001.

HEMGN protein was significantly induced in BM HSPCs 1h following total body irradiation (TBI) exposure (**Figure** **2**A). *Hemgn* mRNA was increased in donor HSPCs from the recipient BM at 6 h post‐BMT (Figure 2B), indicating that HEMGN may contribute to transplantation stress. Since various pro‐inflammatory cytokines could be induced such as tumor necrosis factor *α* (TNF*α*), interleukin 6 (IL‐6), interferon *α* (IFN‐*α*), and IFN‐*γ* under irradiation and BMT conditions, we investigated the expression levels of HEMGN under various cytokines. We found that HEMGN could be induced dramatically by IFN‐*γ* treatment while other cytokines did not affect HEMGN expression significantly (Figure S5, Supporting Information). To assess whether *Hemgn* affects HSPCs regeneration capacity in vivo, a competitive BMT assay was carried out by transplanting WT or *Hemgn* ^−/−^ mice BM (CD45.2^+^) mixed with competitor cells (CD45.1^+^CD45.2^+^) at the indicated ratio into lethally irradiated recipient mice (CD45.1^+^) (Figure 2C). Analysis of the donor chimerism in PB showed that WT BM cells successfully reconstituted recipient's hematopoiesis at 16 weeks post‐BMT, however, none of the recipients transplanted with *Hemgn* ^−/−^ BM cells were reconstituted even when transplanted at a 5‐fold higher dose. When the ratio increased to 10:1, only 2 of 11 recipients transplanted with *Hemgn* ^−/−^ donor cells were reconstituted but with a significant reduction in engraftment (below 5%). Competitive BMT assay using LSK cells showed that *Hemgn* ^−/−^ LSK cells could not engraft and reconstitute hematopoiesis from 4 weeks to 16 weeks post‐BMT (Figure 2D). Noncompetitive BMT assays suggested that WT BM cells (2 × 10^5^) were able to efficiently engraft 4 weeks post‐BMT, but *Hemgn* ^−/−^ BM cells failed to engraft even at cell doses ten times higher than the controls (Figure 2E). 90% of mice transplanted with 5 × 10^5^ WT BM cells survived within 40 days after BMT, while only 10% of mice transplanted with *Hemgn* ^−/−^ BM cells survived (Figure 2F). These data suggest a significant role of *Hemgn* in HSPCs in the engraftment process. When donor cells number was increased to 2 × 10^7^, 100% of the recipients receiving *Hemgn* ^−/−^ BM cells were reconstituted with normal lineage distribution at 16 weeks after BMT (Figure 2E,G), suggesting that *Hemgn* deficiency may not lead to alteration of HSPCs differentiation ability. Transplantation of WT BM cells into *Hemgn* ^−/−^ recipients demonstrated that the engraftment and reconstitution were normal, indicating that *Hemgn* acts in the cells autonomously to maintain HSPCs engraftment activity (Figure 2H).

### 2.3 *Hemgn* Is Required for HSPCs Homing, Survival, and Expansion in Recipient Mice

**Figure 3:**
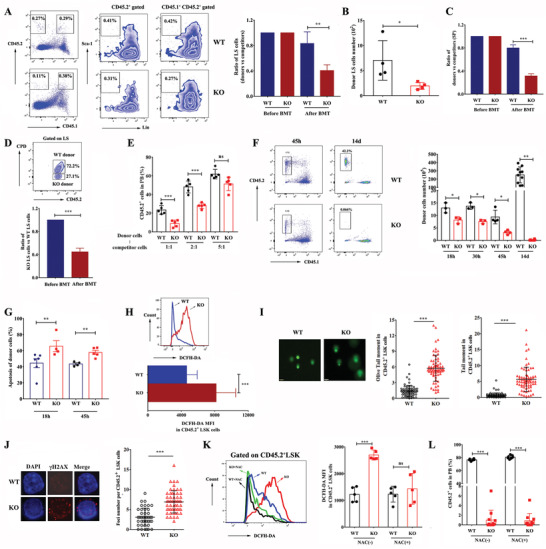
*Hemgn* is required for HSPCs homing, survival and expansion in recipient mice. A) The ratio of Lin^−^Sca‐1^+^ (LS) cells in donor (WT or *Hemgn* ^−/−^) cells versus competitor LS cells and the absolute number of donor derived LS cells B) in the recipients BM in homing assay (*n* = 4 mice per group). The left panel of (A) shows representative flow cytometric plots of homed donor LS cells. Data are representative of two independent experiments. C) The ratio of donor cells versus competitor cells in the recipient's SP was analyzed in homing assay (*n* = 4 mice per group). Data are representative of two independent experiments. D) Cell proliferation dyes (CPD) labeled WT BM cells were mixed with equal number of *Hemgn* ^−/−^ BM cells and transplanted into lethally irradiated CD45.1 recipients. The ratio of *Hemgn* ^−/−^ LS cells (CD45.2^+^CPD^−^) versus WT LS cells (CD45.2^+^CPD^+^) in the recipient's BM was analyzed (*n* = 3 mice per group). The upper panel showed representative flow cytometric plots of donor cells in recipients. Data are representative of two independent experiments. E) WT or *Hemgn* ^−/−^ mice BM mixed with competitor cells at the indicated ratio into the femoral BM cavity of lethally irradiated mice by intrafemoral transplantation. PB was analyzed for donor chimerism at 16 weeks after transplantation (*n* = 5 mice per group). F) WT or *Hemgn* ^−/‐^ BM cells (5 × 10^6^) were transplanted into CD45.1 recipients and the number and apoptosis G) of donor cells in recipients BM were analyzed at the indicated time points. *n* = 3–9 mice. The left panel of (F) shows the representative flow cytometric plots of donor cells in recipients. H) ROS levels in transplanted WT and *Hemgn* ^−/−^ LSK cells from recipients BM at 18 h after transplantation (*n* = 6 mice per group). Data are the pool of two independent experiments. DNA damage analysis of isolated donor LSK cells by quantitation of I) *γ*‐H2AX foci and J) comet assay at 18 h after transplantation. Scale bars: (I) 10 µm; (J) 70 µm. Data are representative of two independent studies. *n* = 3 mice per group. K) Recipients were pre‐treated with NAC (100 mg kg^−1^ day^−1^) for 3 weeks and then transplanted with WT or *Hemgn* ^−/−^ BM cells (1 × 10^7^) accompanied with competitor cells (1 × 10^6^). The ROS levels in donor LSK cells were examined at 18 h after transplantation (*n* = 5 mice per group). L) Recipients were intraperitoneally injected with NAC (100 mg kg^−1^) daily for 3 weeks and then transplanted with WT (*n* = 5 mice per group) or *Hemgn* ^−/−^ BM cells (*n* = 10 mice per group) (1 × 10^7^) accompanied with competitor cells (1 × 10^6^) followed by intraperitoneal injection of NAC (50 mg kg^−1^) daily for 1 week. After that, the recipients were orally administrated with NAC daily by drinking NAC‐containing water (1 mg mL^−1^). The donor chimerism in PB were analyzed at 16 weeks post transplantation. For all graphs, data are presented as mean ± SD. *p < 0.05, **p < 0.01, ***p < 0.001.

Defected HSPCs engraftment could result from reduced HSPCs homing activity, impaired self‐renewal, and decreased proliferation in recipient. We first investigated whether the homing potential of HSPCs was affected by *Hemgn* deficiency. Equal numbers of donor cells and competitor cells were transplanted into lethally irradiated recipient. Competitor BM cells served as internal control in this assay. The ratios of the donor Lin^−^Sca‐1^+^ cells versus competitor cells in both groups were similar before BM transplantation, however, the ratio of the *Hemgn* ^−/−^ Lin^−^Sca‐1^+^ cells versus competitor cells in recipients BM was significant decreased 18 h after transplantation (**Figure** **3**A). Congruently, the absolute number of *Hemgn* ^−/−^ donor‐derived Lin^−^Sca‐1^+^ cells in the recipient mice marrow was also significantly lower compared to donor cells derived from WT mice (Figure 3B), indicating that *Hemgn* deficiency impaired HSPCs homing ability. Similarly, the ratio of *Hemgn* ^−/‐^ HSPCs versus competitor cells in recipients SP was significantly lower at 18 h after transplantation (Figure 3C). In a congenic model, where cell proliferation dyes‐labeled HSPCs were transplanted into lethally irradiated hosts to assess HSPCs homing, a significantly lower number of *Hemgn* ^−/−^ HSPCs homed to BM compared with WT control cells was observed (Figure 3D). These results indicate that the homing capacity of HSPCs is impaired in the absence of *Hemgn*. To confirm, we injected WT or *Hemgn* ^−/−^ mice BM mixed with competitor cells at the indicated ratio into the femoral BM cavity of lethally irradiated mice by intrafemoral transplantation (Figure 3E), which is a strategy to avoid inefficient homing associated with intravenous (IV) HSPCT.^[^ ^20^ ^]^ Analysis of the chimerism in PB for donor cells revealed that the reconstitution ability of donor cells from *Hemgn* ^−/−^ mice was still markedly lower than that of matched WT controls, but intrafemoral transplantation of *Hemgn* ^−/−^ cells significantly improved the engraftment compared with the IV BMT (Figure 3E; Figure S6, Supporting information). When the ratio increased to 5:1, recipients receiving *Hemgn* ^−/−^ BM cells were equivalently reconstituted with normal lineage distribution at 16 weeks post‐BMT (Figure S6, Supporting information), supporting the conclusion that *Hemgn* deficiency reduces HSPCs homing to the BM.

We further investigated donor cells survival and expansion in recipient mice. The results revealed that number of *Hemgn* ^−/−^ donor cells that homed to BM was significantly reduced 45 h compared with 18 h and 30 h post‐BMT; at 14 days, *Hemgn* ^−/−^ donor cells in recipient's BM were almost undetected (Figure 3F). In contrast, the number of WT donor cells in recipient's BM was only slightly reduced at 45 h post‐BMT and then markedly increased at 14 days. In line with these observations, we detected increased apoptosis in transplanted *Hemgn* ^−/−^ donors in BM (Figure 3G) at early stages after transplantation. Using non‐irradiated recipients, we showed that the homing efficiency (Figure S7A, Supporting information) and apoptosis (Figure S7B, Supporting information) of *Hemgn* ^−/‐^ donor cells were comparable to WT donor cells, suggesting that the engraftment defect in *Hemgn* ^−/−^ HSPCs is related to irradiation‐induced microenvironments alteration.

During BMT, irradiation increases the production of reactive oxygen species (ROS), thereby promoting DNA damage in donor HSPCs.^[^ ^5^ ^]^ We found that the ROS levels (Figure 3H) and DNA damage (Figure 3I,J) were significantly elevated in *Hemgn* ^−/−^ donor HSPCs compared with those in WT HSPCs, suggesting that *Hemgn* deficiency caused more serious oxidative stress responses in HSPCs after transplantation.

To examine whether the inhibition of oxidative stress improves engraftment of *Hemgn* ^−/−^ HSPCs, *N*‐acetyl‐l‐cysteine (NAC), an antioxidant which can increase engraftment of HSPCs,^[^ ^21^ ^]^ was used to treat recipient mice as previous described.^[^ ^22^ ^]^ Although NAC reduced the ROS levels significantly in *Hemgn* ^−/−^ donor HSPCs (Figure 3K), it only weakly rescued engraftment defects of *Hemgn* ^−/−^ HSPCs (Figure 3L), demonstrating that oxidative stress contributed little to the function defect of *Hemgn* ^−/−^ HSPCs.

### 2.4 *Hemgn* Deficiency Enhances IFN‐*γ* Signaling Pathway in Transplanted HSPCs and Increases HSPCs Responses to IFN‐*γ* Administration In Vitro and In Vivo

**Figure 4:**
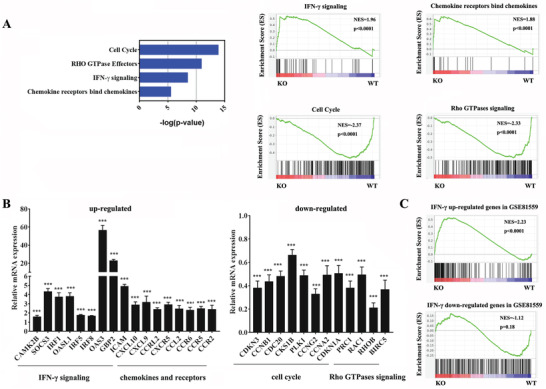
Molecular Signature of *Hemgn* ^−/−^ HSPCs after BMT. A) Reactome pathway analysis of DEGs in transplanted *Hemgn* ^−/−^ HSPCs cells compared with WT HSPCs. B) Validation of indicated genes expression in donor HSPCs cells 6 h after BMT by quantitative RT‐PCR. Data shown are representative of three independent experiments. Error bars indicate SEM. *p < 0.05, ***p < 0.001. C) GSEA of the correlation between DEGs in transplanted *Hemgn* ^−/−^ HSPCs and IFN‐*γ*‐treated HSPCs (GSE81559).

To identify possible mechanisms of the reduced engraftment capability of *Hemgn* ^−/−^ HSPCs, we performed genome‐wide expression analysis using RNA‐seq of purified *Hemgn* ^−/−^ and WT HSPCs from recipient mice at 6h post‐BMT. This revealed 760 differentially expressed genes (DEGs) (325 upregulated and 435 downregulated, *p* < 0.05, 1.5‐fold cut off) (Table S1, Supporting Information). Reactome pathway analysis of DEGs revealed that upregulated genes in *Hemgn* ^−/−^ HSPCs were significantly associated with genes sets related to IFN‐*γ* signaling pathway and chemokine receptors bind chemokines, while the downregulated genes were associated with gene sets related to cell cycle and Rho GTPases signaling (**Figure** **4**A). We validated these genes expression by quantitative PCR analysis (Figure 4B). GSEA analysis revealed a transcriptional signature associated with the IFN‐*γ*‐treated HSPCs (GSE81559) to be the most enriched gene set among upregulated genes in *Hemgn* ^−/−^ HSPCs (Figure 4C), indicating that *Hemgn* deficiency enhanced IFN‐*γ* signaling in HSPCs after transplantation.

**Figure 5:**
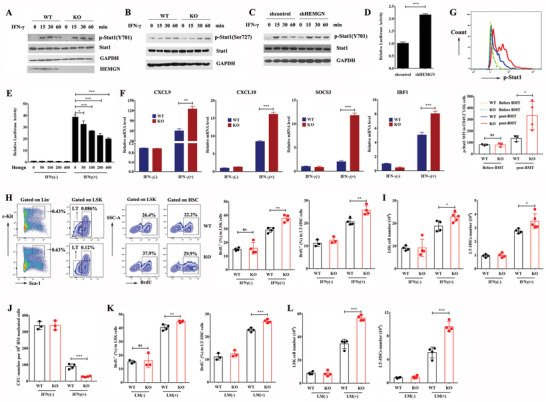
*Hemgn* deficiency increases responses to IFN‐*γ* administration in HSPCs in vitro and in vivo. A) LSK cells were sorted from WT or *Hemgn* ^−/−^ mice and treated with IFN‐*γ* (100 ng mL^−1^) for the indicated time. The tyrosine phosphorylation (p‐Stat1(Y701)) and serine phosphorylation (p‐Stat1(S727) B) of Stat1 were detected. C) *Hemgn* knockdown‐K562 cells or control K562 cells were treated with IFN‐*γ* treatment (100 ng mL^−1^) for the indicated time and the tyrosine phosphorylation of Stat1 was detected. D) GAS‐reporter vector activity in *Hemgn* knockdown‐K562 cells with IFN‐*γ*treatment (100 ng mL^−1^) for 24 h. Data are representative of three independent experiments. Error bars indicate SD. E) GAS‐reporter vector activity in RAW264.7 cells transfected with the indicated doses of *Hemgn* expression vector with or without IFN‐*γ* treatment (100 ng mL^−1^). Data are representative of three independent experiments. Error bars indicate SD. F) Sorted LSK cells from WT or *Hemgn* ^−/−^ mice BM were treated with IFN‐*γ* (100 ng mL^−1^) for 24 h and expression levels of the indicated genes were investigated by quantitative RT‐PCR. *n* = 3 mice per group. Each mouse was performed in triplicate. Error bars indicate SEM. G) Quantification of tyrosine‐phosphorylated Stat1 at residue Y701 in donor WT (*n* = 4 mice) or *Hemgn* ^−/−^ (*n* = 3 mice) LSK cells 12 h after BMT. H) WT or *Hemgn* ^−/−^ mice were intraperitoneally (i.p) administered with 10 µg IFN‐*γ* for 24 h. I) The percentage of proliferating HSPCs (BrdU^+^) and absolute number of HSPCs were analyzed (*n* = 3 or 4 mice per group). Error bars indicate SD. The left panel of (H) showed representative flow cytometric plots. J) CFU assay of BM was performed. Each mouse was performed in triplicate. Error bars indicate SEM. K) WT or *Hemgn* ^−/‐^ mice were i.p injected with 5 × 10^6^ CFU *Listeria monocytogenes* (LM) for 24 h and then the percentage of proliferating HSPCs and absolute number of HSPCs (L) were analyzed (*n* = 3 or 4 mice per group). Error bars indicate SD. For all graphs, *p < 0.05, **p < 0.01, ***p < 0.001.

To access whether *Hemgn* regulates IFN‐*γ* signaling, the phosphorylation of Stat1 induced by IFN‐*γ* was investigated in cultured HSPCs. In WT HSPCs, the level of tyrosine‐phosphorylated Stat1 at residue 701 (p‐Stat1(Y701)) was triggered at 15 min, reached the peak at 30 min, and then markedly declined (**Figure** **5**A). In *Hemgn* ^−/−^ HSPCs, IFN‐*γ*‐triggered p‐Stat1(Y701) was not affected at 15 min but was sustained at higher levels for at least 1 h. No significant difference in IFN‐*γ*‐induced Stat1 serine phosphorylation at residue 727 (p‐Stat1(S727)) was observed between WT and *Hemgn* ^−/−^ HSPCs (Figure 5B). Similar changes of tyrosine‐phosphorylated Stat1 were observed in IFN‐*γ*‐treated K562 cells transfected with *Hemgn* shRNA lentivirus (Figure 5C; Figure S8, Supporting information). IFN‐*γ*‐triggered activation of a luciferase reporter containing a *γ*‐activation sequence (GAS) was enhanced in *Hemgn*‐knockdown K562 cells (Figure 5D). Conversely, enforced expression of HEMGN suppressed IFN‐*γ*‐induced transactivation (Figure 5E; Figure S9, Supporting information). In addition, the expression levels of interferon‐regulated genes were increased in IFN‐*γ* treated *Hemgn^−/−^* HSPCs (Figure 5F). We further investigated the phosphorylation of Stat1 in transplanted donor HSPCs. Flow cytometry analysis revealed that tyrosine‐phosphorylated Stat1 (p‐Stat1(Y701)) staining in *Hemgn* ^−/−^ donor HSPCs was stronger than that in WT donor HSPCs (Figure 5G).

We further examined the response of *Hemgn* ^−/−^ HSPCs to IFN‐*γ* in vivo. In line with previous report,^[^ ^23^ ^]^ a single injection of IFN‐*γ* caused a dramatic increase in the percentage of proliferating HSPCs (Figure 5H) and absolute number of HSPCs (Figure 5I) in WT BM, whereas the changes were significantly augmented in *Hemgn* ^−/‐^ mice. BM cells from IFN‐*γ*‐treated *Hemgn* ^−/−^ mice formed less hematopoietic colonies than those from IFN‐*γ*‐treated WT mice (Figure 5J). A similar alternation of HSPCs was also observed in a mouse model of *Listeria monocytogenes* acute infection (Figure 5K,L; Figure S10, Supporting information), since IFN‐*γ* is necessary to influence the behavior of HSPCs in this model.^[^ ^24^ ^]^

**Figure 6:**
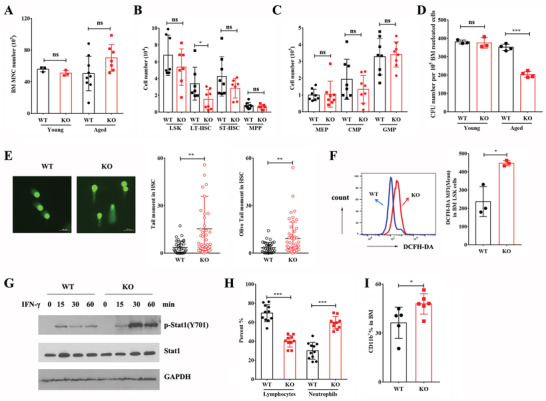
*Hemgn* deficiency leads to depletion of the HSCs compartment over time. A) Total numbers of the BM mononuclear cells and B) HSPCs and C) committed progenitors in the BM of 2‐month‐old (young) (*n* = 3 mice per group) and 28‐month‐old mice (*n* = 7 mice per group). Error bars indicate SD. D) CFU assays of 2‐month‐old (young) (*n* = 3 mice per group) and 28‐month‐old mice BM cells (*n* = 4 mice per group). Each mouse was performed in triplicate. Error bars indicate SEM. E) DNA damage analysis of LT‐HSCs in 28‐month‐old mice BM by comet assay (*n* = 3 mice per group). Error bars indicate SD. F) ROS levels of LT‐HSCs in 28‐month‐old mice BM (*n* = 3 mice per group). Error bars indicate SD. G) LSK cells were isolated from 28‐month‐old mice BM and treated with IFN‐*γ* (100 ng mL^−1^) for the indicated time. The tyrosine phosphorylation of Stat1 was detected. H) Frequencies of neutrophils and lymphocytes in PB of 28‐month‐old mice (*n* = 11 mice in WT group and 10 mice in *Hemgn* ^−/−^ mice group). Data are the pool of two independent experiments with a total of five mice per group. Error bars indicate SD. I) Frequencies of CD11b^+^ cells in BM of 28‐month‐old mice (*n* = 5 mice in WT mice group and *n* = 6 mice in *Hemgn* ^−/−^ mice group). Error bars indicate SD. For all graphs, *p < 0.05, **p < 0.01, ***p < 0.001.

Previous studies suggest that the sustained IFN‐*γ* signaling promotes HSCs depletion in mice,^[^ ^25^, ^26^ ^]^ we postulated that chronically dysregulated IFN‐*γ* signaling in *Hemgn* ^−/−^ mice might lead to diminished self‐renewal and, ultimately, depletion of the HSCs compartment. We examined the BM of young (2‐month‐old) and aged WT and *Hemg*n^−/−^ mice (28‐month‐old). BM mononuclear cells (MNCs) in aged *Hemgn* ^−/−^ mice was comparable to those in aged WT mice (**Figure** **6**A), but the number of phenotypic HSCs in aged *Hemgn* ^−/−^ mice was lower compared with those of age‐matched WT controls (Figure 6B,C; Figure S11, Supporting information). Aged *Hemgn* ^−/−^ BM cells gave rise to about 40% fewer colonies than WT controls (Figure 6D). Moreover, a significant increase in accumulated DNA damage in aged HSCs was noted in *Hemgn* ^−/−^ mice (Figure 6E). Accordingly, the ROS levels in aged *Hemgn* ^−/−^ HSCs were higher than those of age‐matched WT controls (Figure 6F). Consistently, the tyrosine‐phosphorylated Stat1 (p‐Stat1(Y701)) was significantly sustained in aged *Hemgn* ^−/−^ HSPCs with IFN‐*γ* treatment (Figure 6G). In line with the increased HSCs aging, 28‐month‐old *Hemgn* ^−/‐^ mice showed a significant elevation in neutrophils frequency and decrease in lymphocytes frequency in PB (Figure 6H) as well as an increase of CD11b^+^ cells frequency in BM (Figure 6I) compared with the age‐matched WT controls. No death was observed in *Hemgn* ^−/‐^ mice over time. These findings suggest that *Hemgn* deficiency can lead to depletion of the HSCs compartment and increased HSCs aging over time.

### 2.5 Engraftment Defect of *Hemgn* ^−/−^ HSPCs Is Rescued by Blocking IFN‐*γ* Signaling Pathway

**Figure 7:**
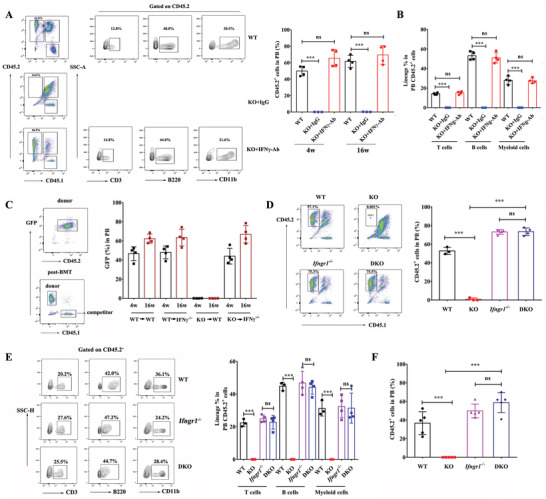
Engraftment defect of *Hemgn* ^−/−^ HSPCs is rescued by blocking IFN‐*γ* signaling pathway. A) WT or *Hemgn* ^−/−^ BM cells (2 × 10^6^) with competitor cells (1 × 10^6^) were transplanted into CD45.1 recipients followed by treatment with anti‐IFN‐*γ* monoclonal antibody and the donor chimerism in PB was analyzed at the indicated time points (*n* = 4 mice per group). The left panel showed representative flow cytometric plots of recipients at 16 weeks after transplantation. B) The trilineage differentiation in PB was analyzed at the indicated time after transplantation. C) *Hemgn* ^−/−^‐GFP or GFP transgenic mice BM cells were transplanted into IFN‐*γ* ^−/−^ mice and the donor chimerism in PB was analyzed at 16 weeks after transplantation. The left panel showed representative flow cytometric plots of donor cells and recipient mice transplanted with donor cells (*n* = 4 mice per group). D) BM cells from WT (*n* = 3 mice), *Hemgn* ^−/−^(KO) (*n* = 3 mice), *Ifngr*1^−/−^ (*n* = 4 mice), or *Hemgn* ^−/−^ *Ifngr*1^−/−^(DKO) (*n* = 4 mice) (2 × 10^6^) were transplanted into irradiated recipients (CD45.1) in the presence of competitor cells (1 × 10^6^). E) The donor chimerism and trilineage differentiation in PB were analyzed at 16 weeks after transplantation. The left panel showed representative flow cytometric plots of trilineage differentiation. F) Noncompetitive BMT assay of BM cells from the indicated mice models at 4 weeks post‐BMT (*n* = 5 mice per group). For all graphs, data are presented as mean ± SD. ***p < 0.001.

Based on the results obtained above, we asked if the abnormal activation of IFN‐*γ* signaling might be the major cause of engraftment defect of *Hemgn* ^−/−^ HSPCs. We blocked IFN‐*γ* signaling by injection with anti‐IFN‐*γ* monoclonal antibody to neutralized serum IFN‐*γ* and the results showed that IFN‐*γ* neutralization fully rescued the engraftment defect of *Hemgn* ^−/‐^ HSPCs, but injection of IgG failed to improve the engraftment of *Hemgn* ^−/−^ BM cells (**Figure** **7**A). Lineage distribution analysis indicated that the trilineage differentiation of BM cells from anti‐IFN‐*γ* antibody‐treated *Hemgn* ^−/‐^ recipients was normal (Figure 7B). To further confirm the role of IFN‐*γ* signaling, we generated *Hemgn* ^−/−^‐GFP transgenic mice and transplanted *Hemgn* ^−/−^‐GFP BM to IFN‐*γ* gene knockout mice (IFN‐*γ* ^−/−^) with competitor cells. The results showed that *Hemgn* ^−/−^ HSPCs successfully engrafted and reconstituted the hematopoiesis in IFN‐*γ* ^−/−^ mice (Figure 7C), and the engraftment was similar to that in WT donor‐transplanted mice.

IFN‐*γ* uses a heterodimeric receptor consisting of IFNGR1 and IFNGR2 chains. We generated double mutant mice lacking both *Hemgn* and the *Ifngr1* (DKO) and performed competitive BMT experiments. Chimerism analysis in PB at 16 weeks post‐BMT indicated that DKO donor cells successfully reconstituted the hematopoiesis (Figure 7D). Lineage distribution analysis showed intact differentiation of trilineage from DKO PB (Figure 7E), BM (Figure S12, Supporting Information), and SP (Figure S13, Supporting Information). The similar result was obtained in noncompetitive BMT assay (Figure 7F). These data suggest that the engraftment defect of *Hemgn* ^−/−^ HSPCs might be due to dysregulated IFN‐*γ* signaling in *Hemgn* ^−/−^ HSPCs.

### 2.6 *Hemgn* Regulates IFN‐*γ* Signaling Pathway through Modulating T‐Cell Protein Tyrosine Phosphatase TC45 Activity

**Figure 8:**
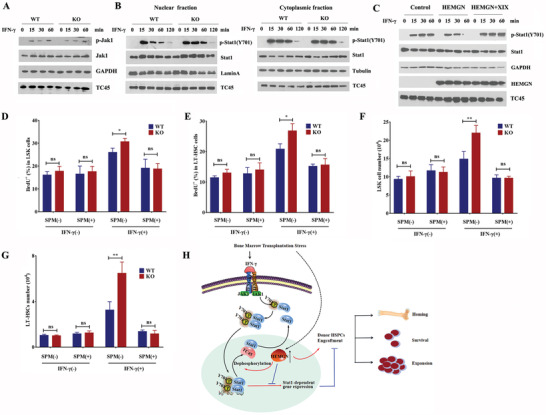
*Hemgn* regulates IFN‐*γ* signaling pathway through modulating TC45 activity. A) LSK cells were sorted from WT or *Hemgn* ^−/−^ mice BM and treated with IFN‐*γ* (100 ng mL^−1^) for the indicated time. Total cell lysates were prepared and the phosphorylation of Jak1 was detected. B) Nuclear and cytoplasmic extracts were isolated from LSK cells treated with IFN‐*γ* (100 ng mL^−1^) for the indicated time and the tyrosine phosphorylation of Stat1 was detected. C) HepG2 cells were transiently transfected with HEMGN for 36 h and pretreated with PTP inhibitor XIX (2 µm) for 2 h. Then the cells were treated with IFN‐*γ* (50 ng mL^−1^) for the indicated time and the tyrosine phosphorylation of Stat1 and TC45 expression were detected. D,E) WT and *Hemgn* ^−/−^ mice were treated with spermidine (SPM) for 3 days followed by i.p injection with 10 µg IFN‐*γ* for 24 h. F,G) The proliferating HSPCs and absolute number of HSPCs were analyzed (*n* = 3 or 4 mice per group). For all graphs, data are presented as mean ± SD. *p < 0.05, **p < 0.01. H) Model depicting the critical role of *Hemgn* in donor HSPCs engraftment during BMT. *Hemgn* is induced by irradiation or BMT stress which in turn regulates the key aspects of the transplanted HSPCs through promoting TC45‐medicated Stat1 dephosphorylation, which deactivates IFN‐*γ* signaling, resulting in protection of HSPCs from transplantation stress.

IFN‐*γ* triggers JAK‐STAT signaling pathway through binds to its receptors,^[^ ^27^ ^]^ leading to phosphorylation of JAK1, followed by tyrosine phosphorylation of Stat1. Our result showed that *Hemgn* deficiency had no effect on IFN‐*γ* receptors expression (Figure S14, Supporting information) and IFN‐*γ* induced JAK1 phosphorylation in HSPCs (**Figure** **8**A), indicating that *Hemgn* does not affect the upstream events of IFN‐*γ* pathway. Given that HEMGN is mainly located in nucleus, and *Hemgn* deficiency prolonged Stat1 tyrosine phosphorylation in IFN‐*γ*‐treated HSPCs, we hypothesized that the molecular target of *Hemgn* is closely linked to modulate Stat1 phosphorylation level in nucleus. Indeed, we demonstrated that the p‐Stat1(Y701) was sustained at higher levels from 30 min to 1 h after IFN‐*γ* treatment in the nuclear fraction of *Hemgn* ^−/−^ HSPCs compared with that in IFN‐*γ* treated WT HSPCs, although no obvious difference was observed at 15 min (Figure 8B). In contrast, the levels of p‐Stat1(Y701) in cytoplasmic fraction of HSPCs was not affected by *Hemgn* deficiency (Figure 8B).

Previous studies demonstrate that dephosphorylation of Stat1 in nucleus is a key mechanism for inactivation of Stat1 activity.^[^ ^28^ ^]^ TC45 is the major nuclear Stat1 protein tyrosine phosphatase (PTPase),^[^ ^29^ ^]^ we therefore speculated that HEMGN may be linked to modulate TC45‐medicated Stat1 dephosphorylation. We found that overexpression of HEMGN led to an diminished p‐Stat1(Y701) levels in HepG2 cells treated with IFN‐*γ*, however, this effect was rescued by administration of PTPase inhibitor XIX, a selective inhibitor of TC45,^[^ ^30^ ^]^ suggesting that HEMGN might regulate Stat1 dephosphorylation through TC45 (Figure 8C). Notably, the levels of TC45 expression were not affected by *Hemgn* deficiency or overexpression (Figure 8A,C).

Furthermore, spermidine (SPM), a selective agonist of TC45,^[^ ^31^ ^]^ was employed to investigate the effect of *Hemgn* on IFN‐*γ* sensitivity of HSPCs in vivo. Administration of spermidine did not affect the behavior of HSPCs in both genotype mice in the absence of IFN‐*γ* (Figure 8D–G). Upon IFN‐*γ* treatment, spermidine administration significantly rescued the exacerbated response to IFN‐*γ* in *Hemgn* ^−/−^ HSPCs (Figure 8D–G). These results indicate that *Hemgn* deletion accelerates HSPCs response to IFN‐*γ* in *Hemgn* ^−/−^ mice via suppressing TC45 activity.

## 3 Discussion

Although *Hemgn* has been reported to be an important regulator for hematopoietic cells, the role of endogenous *Hemgn* in HSPCs has not been investigated yet. In this study, we used genetic tools to identify a novel role for *Hemgn* as a critical regulator of HSPCs function in response to transplantation stress. Our data show that *Hemgn* is markedly induced in HSPCs during BMT to regulate key aspects of the transplanted HSPCs through promoting TC45‐medicated Stat1 dephosphorylation, which deactivates IFN‐*γ* signaling and protects HSPCs from transplantation stress (Figure 8H). Our findings provide novel insight into how HSPCs combat transplantation stress to successfully replenish a damaged hematopoietic system. Although previous studies report that IFN‐*γ* signaling is essential for embryonic hematopoietic stem and progenitor cell production,^[^ ^32^, ^33^ ^]^ *Hemgn* loss had limited effects on steady hematopoiesis in young mice. Given that phenotypic long‐term repopulating HSCs are reportedly present at normal frequencies in the adult BM of IFN‐*γ*‐deficient mice,^[^ ^23^ ^]^ we suppose that there might be a threshold level of IFN‐*γ* stimulation above which *Hemgn* is functionally important. Indeed, we found that old *Hemgn* ^−/−^ mice displayed decreased HSCs number and increased ROS level and DNA damage in HSCs, suggesting that chronically dysregulated IFN‐*γ* signaling in *Hemgn* ^−/−^ mice might lead depletion of the HSCs compartment and increased HSCs aging.

We found that the most transplanted *Hemgn* ^−/−^ HSPCs did not effectively home to the BM cavity post‐transplant. Transplantation of *Hemgn* ^−/−^ HSPCs by intrafemoral injection significantly improved the engraftment, suggesting that impairment of homing activity is a key event in the BMT defect caused by *Hemgn* deficiency. Additionally, *Hemgn* ^−/−^ HSPCs exhibited impaired expansion and increased apoptosis in recipients after BMT. Transcriptome profiling analysis revealed that *Hemgn* disruption decreased the expression of a set of genes associated with HSPCs homing, cell cycle and proliferation, and survival such as RAC1, CKS1B, CDKN1A, Lkb1, and BIRC5,^[^ ^34^, ^35^, ^36^, ^37^, ^38^ ^]^ indicating that multiple genes and mechanisms are involved in the function defect of *Hemgn* ^−/−^ HSPCs. *Hemgn* is induced by irradiation and transplantation stress indicating a regulatory feedback mechanism. Transplanted *Hemgn* ^−/−^ HSPCS in non‐irradiated recipients display normal homing activity and survival, suggesting that the loss of engraftment ability in *Hemgn* ^−/−^ HSPCs is related to irradiation‐induced microenvironments alteration.

*Hemgn* ^−/−^ HSPCs displayed hypersensitivity to IFN‐*γ* treatment in vitro and in vivo, indicating that *Hemgn* is a negative regulator of IFN‐*γ* signaling in HSPCs. IFN‐*γ* has been demonstrated pleiotropic influences on HSPCs proliferation, quiescence, reconstitution, mobilization and differentiation.^[^ ^23^, ^25^, ^39^, ^40^, ^41^, ^42^, ^43^ ^]^ IFN‐*γ*‐deficient HSCs are more quiescent and perform better in transplantation assays, conversely, transplantation of IFN‐*γ*‐treated HSC into lethally irradiated mice result in reduced short‐term engraftment and long‐term reconstituting activity.^[^ ^23^, ^41^ ^]^ IFN‐*γ* and Stat1 arrest hematopoietic cells migration through modulating RAC/CDC42 pathways.^[^ ^44^ ^]^ Increased serum levels of IFN‐*γ* and CXCL9 represent potential biomarkers useful for early diagnosis of graft failure in patients.^[^ ^45^ ^]^ HSCT for patients with gain‐of‐function mutation of Stat1 has significant risk of secondary graft failure and death.^[^ ^46^ ^]^ These studies clearly show that tight control of IFN‐*γ* signaling is critical for the maintenance of HSCs function and suppression IFN‐*γ* signaling may improve engraftment of HSPCs during BMT. We demonstrated that blocking IFN‐*γ* signaling pathway by genetic or antibody inhibition almost completely rescued the engraftment defect of *Hemgn* ^−/−^ HSPCs in mice, supporting that negative regulation of IFN‐*γ* signaling is contributed to protection of HSPCs from transplantation stress by *Hemgn*. Our findings is consistent with previous findings that loss of feedback inhibition of IFN‐*γ* signaling by immunity‐related p47 GTPase (Irgm1) or adenosine deaminase acting on RNA‐1 (ADAR1) deficiency results in severe function defect of HSPCs.^[^ ^26^, ^47^ ^]^

The dephosphorylation of Stat1 by TC45 is a key event in Stat1‐induced transcriptional inactivation and termination.^[^ ^29^ ^]^ The evidences presented here suggest that the negative regulation of IFN‐*γ* signaling by *Hemgn* is associated with this process. *Hemgn* does not affect the upstream events of IFN‐*γ* pathway but prolonged nuclear Stat1 tyrosine phosphorylation. Inactivation of TC45 by pharmaceutical inhibitor effectively reversed the inhibition effect of HEMGN on IFN‐*γ*‐induced p‐Stat1(Y701) in HepG2 cells. Furthermore, spermidine (a selective agonist of TC45) treatment rescued the exacerbated response of *Hemgn* ^−/−^ HSPCs to IFN‐*γ* in mice. These results suggest that *Hemgn* deletion accelerates HSPCs response to IFN‐*γ* via suppressing TC45 activity. Given that *Hemgn* deficiency does not alter TC45 expression and HEMGN can interact with a variety of nuclear proteins,^[^ ^14^, ^15^, ^48^ ^]^ the investigation of interactions between HEMGN and Stat1/TC45 may help to explain the exact mechanism by which HEMGN regulates TC45‐medicated Stat1 dephosphorylation.

Although present studies focused on the murine *Hemgn*, several evidences point out a potential role of *Hemgn* in humans as well. First, *Hemgn* is induced in human cells after irradiation or hypoxia exposure.^[^ ^18^, ^19^ ^]^ Second, *Hemgn* is involved in regulation of IFN‐*γ* response in human hematopoietic cells K562, as evidenced by that overexpression of *Hemgn* suppressed IFN‐*γ*‐induced GAS‐luciferase activity and knockdown of *Hemgn* enhanced IFN‐*γ*‐triggered transactivation activity. Third, microarray analysis reveal that knockdown of *Hemgn* in human CD34^+^ cells increases the expression of IFN‐*γ*‐inducible genes.^[^ ^17^ ^]^ Fourth, in a global transcriptome analysis of CD34^+^ cells from severe aplastic anemia patients, which the function of HSPCs is impaired by intrinsic IFN‐*γ* inhibition, revealed significant down‐regulation of *Hemgn*.^[^ ^49^ ^]^ Most importantly, our previous studies show that overexpression of HEMGN enhances the proliferative potential of human cord blood CD34^+^ cells, increases survival, prevents cell apoptosis and promotes their repopulating capacity.^[^ ^17^ ^]^ Future studies will be necessary to determine whether *Hemgn* may be considered as therapeutic targets for patients with IFN‐*γ*‐mediated BM failure or those with extensive IFN‐*γ* exposure such as during BMT stress and chronic infections.

In summary, our results provide new insights into the mechanisms employed by HSPCs to withstand the BMT stress and may be used to improve conditioning regimens and engraftment during BMT.

## 4 Experimental Section

### Mice

*Hemgn*‐deficient (*Hemgn* ^−/−^) mice were successfully constructed using Zinc Finger Nuclease technology (ZFNs) in a C57BL/6 (CD45.2) background. Interferon‐gamma receptor 1‐deficient *(Ifngr1* ^−/−^) mice in C57BL/6 (CD45.2) background were purchased from Jackson Laboratory (Bar Harbor, ME). *Hemgn* ^−/−^ and *Ifngr1* ^−/−^ strains were intercrossed to produce mice that were homozygous for disruptions at the *Hemgn* and *Ifngr1* loci (*Hemgn* ^−/‐^ *Ifngr1* ^−/−^, DKO). *Hemgn* ^−/−^ and GFP transgenic mice (C57BL/6‐TgN(act‐EGFP)OsbC14‐Y01‐FM131) were similarly intercrossed to produce mice that were homozygous for *Hemgn* mutation that expressed the GFP (*Hemgn* ^−/−^‐GFP). In all experiments, male and female mice between 8 and 10 weeks of age were used in the studies. Genetically modified mice were systematically compared to their sex‐, age‐, and weight‐matched wild type (WT) littermates. All mice were housed in individually ventilated cages under specific pathogen‐free conditions at the animal facility of the authors’ institute with a 12 h light‐dark cycle and allowed free access to food and water. All animal experiments were reviewed and approved by the Animal Ethics Committee of the Beijing Institute of Lifeomics.

### Bone Marrow Transplantation Experiments

For competitive transplantation, donor cells were mixed with 1 × 10^6^ freshly isolated competitor BM cells (CD45.1^+^CD45.2^+^) and then transplanted into recipients previously irradiated with a split dose of 9Gy (4.5Gy+4.5Gy) through tail vein injection. For non‐competitive transplantation, donor cells were injected into recipients without competitor cells. Engraftment was measured at the time points indicated and analyzed for PB chimerism and multilineage engraftment. For intrafemoral transplantation, recipients were anesthetized with 1% pelltobarbitalum natricum (Sinopharm Chemical Reagent Co., Ltd.), and the joint between the femur and tibia in one leg was surgically exposed. Donor cells mixed with 2 × 10^5^ freshly isolated competitor cells were injected into the femur medullary cavity near the medial epicondyle. The insertion hole was immediately sealed with bone wax, and the skin was closed by wound clips.

### Flow Cytometry

Antibodies used for flow cytometry analysis or cell sorting were listed in Supporting Information Table S2, Supporting Information. LSRFortessa (BD) were used for flow cytometric analysis and FACSAria III (BD) for cell sorting. HSPCs were identified as viable LSK (Lin^−^Sca‐1^+^c‐Kit^+^) as previously described.^[^ ^50^ ^]^ Before staining, red blood cells were eliminated by RBC Lysis Buffer (Biolegend). For HSPCs sorting, Lin^−^ cells were enriched using EasySep Mouse Hematopoietic Progenitor Cell Enrichment Kit (Cat.19756, STEMCELL Technologies). Then the enriched Lin^−^ cells were stained with relative surface markers and sorted for further in vivo and in vitro investigations.

### Homing Assay

Homing assay was performed as previous described^[^ ^51^ ^]^ with some modifications. Briefly, BM cells prepared from *Hemgn* ^−/−^ or WT control mice were mixed with competitor BM cells (CD45.1^+^CD45.2^+^), and then were injected into lethally irradiated recipient mice. Eighteen hours after injection, the ratio of Lin^−^Sca‐1^+^ (LS) cells in injected test versus competitor BM cells (percentage of CD45.2^+^ LS cells to percentage of CD45.1^+^CD45.2^+^ LS cells) was analyzed in BM cells. In addition, homing assays were also performed with Cell Proliferation Dyes (CPD)‐labeled donor cells. CPD staining was performed according to the manufactures’ instructions. Single cell suspensions of bone marrow cells to be injected were washed once resuspended in PBS (1.0–2.0 × 10^7^ cells mL^−1^). To this suspension, the same volume of a 10 µm solution of Cell Proliferation Dye eFluor 670(CPD,65‐0840, eBioscience,) was added to give a final concentration of 5 µm CPD. 10 µm CPD solution was prepared by dilute 5 mm stock solution (dissolved in anhydrous DMSO) in PBS (pre‐warmed to room temperature). The cells were then incubated at 37 °C in the dark for 10 min. Further labeling of the CPD was stopped by addition of 5 volumes of cold complete media (containing 20% serum in DMEM media) and incubate on ice for 5 min. The suspension was washed three times with complete media and finally resuspended in PBS. The CPD‐labelled cells and other BM cells were counted respectively by FACS using 123 count beads (1234‐42, eBioscience). These cells were mixed in proportion to get the final concentration of 5 × 10^7^ cells mL^−1^ and injected intravenously with the volume of 200 uL.

### RNA‐Seq and Analysis

LSK cells from primary recipient mice BM (three biological replicates) at 6 h post‐BMT were sorted using BD FACSAria III. Total RNA was isolated using the QIAGEN RNeasy Micro Kit (QIAGEN). Smart‐Seq2 method was used to produce cDNA. The cDNA production was checked by Qubit 3.0 Flurometer and Agilent 2100 Bioanalyzer to ensure the expected production with length around 1–2 kbp. Then the cDNA was sheared randomly by ultrasonic waves for Illumina library preparation protocol including DNA fragmentation, end repair, 3′ ends A‐tailing, adapter ligation, PCR amplification, and library validation. After library preparation, PerkinElmer LabChip GX Touch and Step OnePlus Real‐Time PCR System were introduced for library quality inspection. Qualified libraries were then loaded on Illumina Hiseq platform for PE150 sequencing at the ANNOROAD GENOME Co., Ltd. (Beijing, CN) following the vendor's recommended protocol. Only transcripts with FKPM greater than 1 at least one samples were selected for differential testing. Transcripts with *p* < 0.05 and fold change ±1.5 were considered differentially expressed. The enrichment analysis of pathways was performed using reactome pathway database (https://reactome.org). GSEA analysis with GSE81559 (IFN‐*γ*‐dependent genes in HSPCs) was carried out using GSEA 4.0.0 and MsigDB 7.0.^[^ ^52^ ^]^ The accession number for the RNA‐seq data reported in this paper is GEO: GSE146949.

### IFN‐*γ* and IFN‐*γ* Antibody Treatment

For IFN‐*γ* treatment, 10 µg/mouse recombinant murine IFN‐*γ* (Cat.315‐05, PeproTech, RockHill, NJ), was injected intraperitoneally into *Hemgn* ^−/−^ and WT mice. For IFN‐*γ* neutralizing antibody treatment, mice were injected intraperitoneally with 200 µg of IFN‐*γ* neutralizing antibody (Cat.505847, Biolegend) or control antibody (Cat.400432, Biolegend) in 100 µL PBS twice per week for 3 weeks after BMT.

### Western Blotting

To obtain whole‐cell extracts, cells were washed with PBS and incubated for 20 min in cold lysis buffer containing freshly added protease inhibitors (Beyotime). Protein concentrations were determined using BCA protein assay (Beyotime). Total protein (10 mg) was separated by SDS–PAGE and transferred to polyvinylidene difluoride membranes (Millipore). Membranes were probed with specific primary antibodies, antibody–protein complex detected by horseradish peroxidase‐conjugated secondary antibodies and enhanced chemiluminescence (ECL) exposed (Pierce).

### Spermidine Treatment

For spermidine (Cat.S3569, Selleckchem) treatment experiments, mice were received intraperitoneal administration of spermidine dissolved in PBS (10 mg kg^−1^, twice daily, for 3 days) prior to IFN‐*γ* treatment.

### Measurement of ROS

Indicated cells were incubated with 1 mm 5‐(and‐6)‐carboxy‐29,7 9 ‐Dichloro fluorescein diacetate (DCFH‐DA, S0033; Beyotime) for 30 min at 37 °C in the dark. Then, cells were stained for other markers when needed and assayed with a flow cytometer.

### Statistical Analysis

Unless otherwise indicated, data in all figures are expressed as the mean ± SD. Statistical details relevant to each experiment including *n* and number of times each experiment was repeated are listed in the figure legends. For comparisons between 2 groups, 2‐tailed Student's‐*t* tests were performed. For multiple group (more than 2 groups) comparisons, One‐way ANOVA tests were performed. In the mouse survival experiments, survival rates were plotted in Kaplan–Meyer survival curves and analyzed with the log‐rank non‐parametric test. Statistical significance is indicated in figures as follows: *p < 0.05; **p < 0.01; ***p < 0.001. Data values were analyzed and graphed using GraphPad Prism software version 7.

## Conflict of Interest

The authors declare no conflict of interest.

## Author Contributions

K.Z., J.‐F.L., X.‐M.D., and R.‐H.Y.: Designed research, performed research, and analyzed data. Y.‐X.Z., H.‐Y.G., F.‐J.X., R.G., Q.W., Y.‐Q.Z., M.Y., H.C., H.‐M.N., and C.‐B.Z.: Performed research. X.L.: Analyzed data. X.‐M.Y.: Designed research, analyzed data, and wrote the paper. C.‐Y.L.: Designed research, performed research, analyzed data, and wrote the paper.

## Supporting information

Supporting Information

Supplemental Table 1

Supplemental Table 2

## Data Availability

The data that support the findings of this study are available from the corresponding author upon reasonable request.
