# Supplementary material for: Hemgn Protects Hematopoietic Stem and Progenitor Cells Against Transplantation Stress Through Negatively Regulating IFN‐γ Signaling
Source: Adv Sci (Weinh). 2021 Dec 19;9(5):2103838. doi: 10.1002/advs.202103838 (PMC8844507; doi:10.1002/advs.202103838)
Supplement: Supplementary file 1 — Supporting Information [file ADVS-9-2103838-s003.pdf]

ADVANCED SCIENCE
Open Access

Supporting Information

for *Adv. Sci.*, DOI: 10.1002/advs.202103838

Hemgn protects hematopoietic stem and progenitor cells against transplantation stress through negatively regulating IFN-γ signaling

Ke Zhao, Jin-Fang Liu, Ya-Xin, Zhu, Xiao-Ming Dong, Rong-Hua Yin, Xian Liu, Hui-Ying Gao, Feng-Jun Xiao, Rui Gao, Qi Wang, Yi-Qun Zhan, Miao Yu, Hui Chen, Hong-Mei Ning, Cai-Bo Zhang, Xiao-Ming Yang, and Chang-Yan Li**

Supporting information

***Hemgn* protects hematopoietic stem and progenitor cells against transplantation stress through negatively regulating IFN-γ signaling**

Ke Zhao, Jin-Fang Liu, Ya-Xin, Zhu, Xiao-Ming Dong, Rong-Hua Yin, Xian Liu, Hui-Ying Gao, Feng-Jun Xiao, Rui Gao, Qi Wang, Yi-Qun Zhan, Miao Yu, Hui Chen, Hong-Mei Ning, Cai-Bo Zhang, Xiao-Ming Yang, Chang-Yan Li

This file contains supplementary materials and methods, and 14 supplementary figures.

Supplementary Materials and Methods

Cell isolation

BM cells were flushed from intact femurs and tibia, and spleens were mashed with 2mL disposable syringe plunger to generate single cell suspension. Collection of the cells was performed in HDMEM media with 5% FBS or PBS with 0.5% BSA, and filtered through a 70-μm strainer. Unless otherwise stated, all cell numbers in this study were standardized as total counts per mouse or per spleen.

Colony forming unit assay

20,000 bone marrow nucleated cells were cultured in triplicated in Methocult M3434 (Stem Cell Technologies), and colonies were scored by microscopy.

Serial replating

30000 BM nucleated cells from WT and *Hemgn*$^{-/-}$ mice were plated in Methocult medium for one week. After that time, colonies were counted, harvested and 30,000 cells were replated every passage.

Cell cycle analysis

For *in vivo* cell-cycle analysis with BrdU, the FITC BrdU Flow kit (BD Pharmingen) was used according to the manufacturer's protocol, and analyzed by flow cytometry. For *in vivo* labeling with BrdU, mice were intravenously injected with BrdU (100 mg/kg) twice at 12h and 3h before euthanization. For Ki67 staining, cells were fixed using BD Cyto/Cytoperm™ Fixation/Permeabilization Solution Kit (BD Pharmingen) and staining with Ki67 antibody.

Comet assay

The alkaline comet assay was performed to evaluate the degree of DNA damage using OxiSelect™ Comet Assay Kit (CELL BIOLABS) according to the manufacturer's instructions. Briefly, cells were mixed with Comet Agarose at 1:10 ratio (v/v), titrate to mix, and immediately pipette 75µL/well onto the OxiSelect™ Comet Slide. The prepared samples were lysed for 40min at 4℃ in the dark and rinsed 3 times in cold 70% Ethanol. The samples were then subjected to electrophoresis, stained with Vista Green DNA Dye and observed and photographed using a confocal fluorescence microscope. Olive tail moment and tai moment were quantitated.

Quantitative reverse transcriptase-polymerase chain reaction

Total RNA was isolated with Trizol reagent (Invitrogen) according to the manufacturer's recommendation. First-strand cDNA was synthesized from 1 ug of total RNA in a 10 μL reaction with oligo dT primer using the Transcriptor High Fidelity cDNA Synthesis Kit (Roche). Real-time PCR reaction was performed with the ExTaq SYBR Green Supermix (Takara) using LightCycler96 Real-Time PCR Detection System (Roche). Each PCR reaction generated a specific amplicon, as demonstrated by melting-temperature profiles of final products (dissociation curve analysis). No PCR products were observed in the absence of template. The amount of target RNAs were normalized to the amount of endogenous control, glyceraldehyde-3-phosphate dehydrogenates (GAPDH). Gene expression quantities were measured using the relative Ct method. The primers used in this study were shown in Supplementary information Table S3.

Immunofluorescence

Indicated cells were placed on poly-L-lysine coated slides, fixed with 4% paraformaldehyde, permeabilized by 0.3% Triton-X 100 and blocked by 2% BSA-PBS. γ-H2AX antibody (AP0099, ABclonal) and was used at 1:200 in the blocking solution. The secondary antibody conjugated with fluorescence (Santa Cruz) was incubated for 30min. Lastly, DAPI was used for nuclear staining. The slides were then visualized and photographed using a confocal fluorescence microscope (Ultra VIEW Vox PerkinElmer).

Luciferase reporter assay

For GAS activity assay, K562 cells or RAW264.7 cells were transfected with the GAS-Luc, pRL-TK-Luc reporter using jetPRIME® (Polyplus Transfection) according to the manufacturer's instructions. pRL-TK-Luc reporter was used as an internal control. Twenty-four hours after transfection, cells were treated with 100ng/mL IFN-γ for 12h and then luciferase activities were measured with the Dual Luciferase Assay system (Promega) according to the manufacture's instructions. All transfection and reporter assay were performed independently at least three times.

NAC administration. Before transplantation, mice were treated daily for 3 weeks with antioxidant N-acetyl-L-cysteine (NAC; 100mg/kg body weight; Sigma-Aldrich) by intraperitoneal injection. After transplantation, NAC was given to recipient mice intraperitoneally at a dose of 50 mg/kg body weight every day for 1 weeks or in drinking water with 1 mg/mL NAC for the duration of the entire experiment.

Apoptosis analysis. For apoptosis analysis, cells were washed and stained using an Annexin V Apoptosis Detection Kit (KeyGEN BioTECH or eBioscience). All the

samples were then analyzed on a flow cytometer.

Supplementary Figures

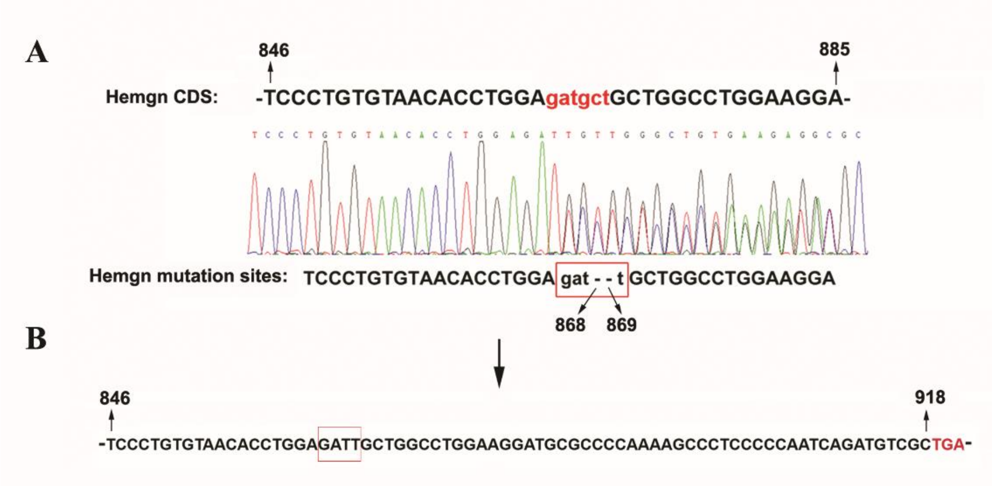

Figure S1. Validation of *Hemgn* knockout mice by sequencing of the mutant sites in cDNA region. The editing results in a loss of 2 bases which locates at 868-869 of *Hemgn* CDS sequence and introduces a premature stop codon at 919-921. (A) Sequence of *Hemgn*$^{+/-}$ heterozygous alleles. (B) Sequence of the mutant allele after editing.

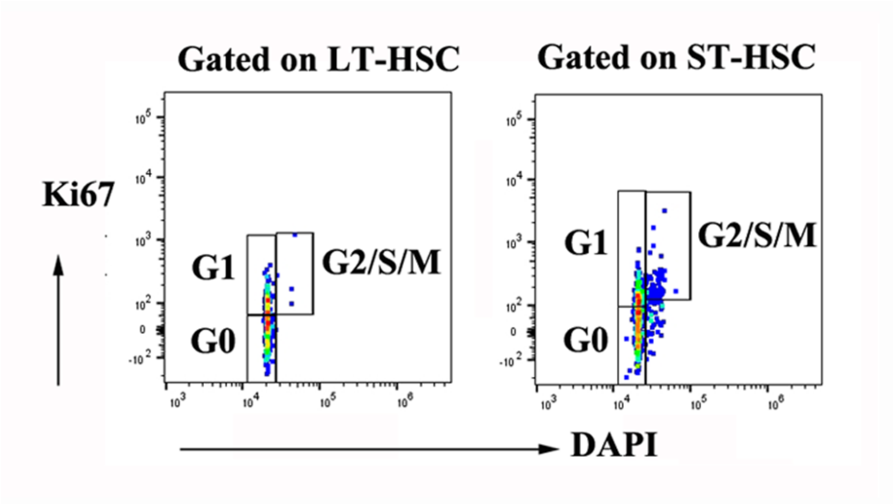

Figure S2 Representative flow cytometric plots for cell cycle analysis of LT-HSCs and ST-HSCs using DAPI and Ki-67 staining.

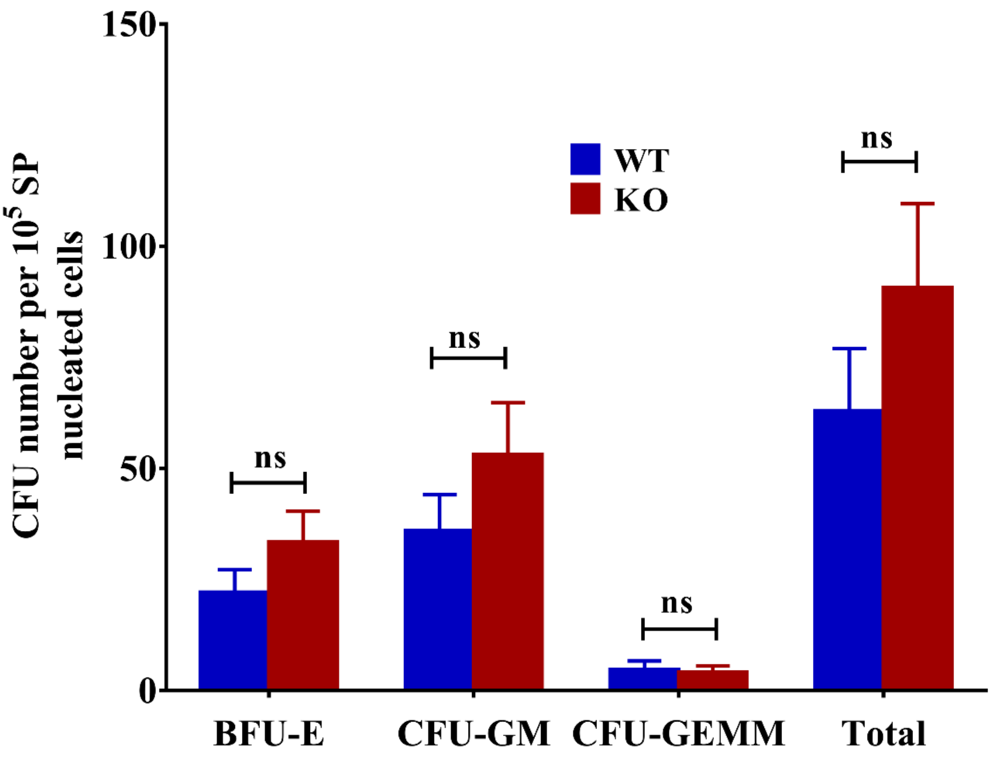

Figure S3. Colony forming unit assays showed normal differentiation and proliferation of *Hemgn*$^{-/-}$ spleen (SP) cells (n=5 mice per group). Data are the pool of 2 independent experiments with a total of 5 mice per group. Each mice were performed in triplicate. Error bars indicate SEM. *P < 0.05.

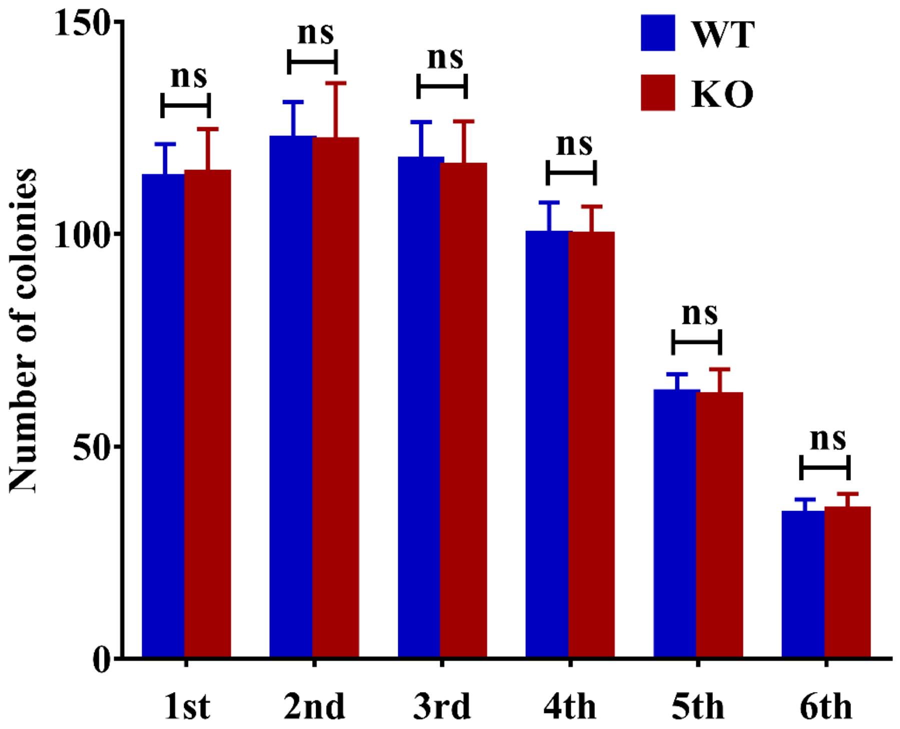

Figure S4. Serial replating assay to compare the self-renew capacity of BM cells from WT and *Hemgn*$^{-/-}$ mice (n=3 mice per group). Data are representative of 2 independent experiments. Each mice were performed in triplicate. Error bars indicate SEM. $^{*}P < 0.05$.

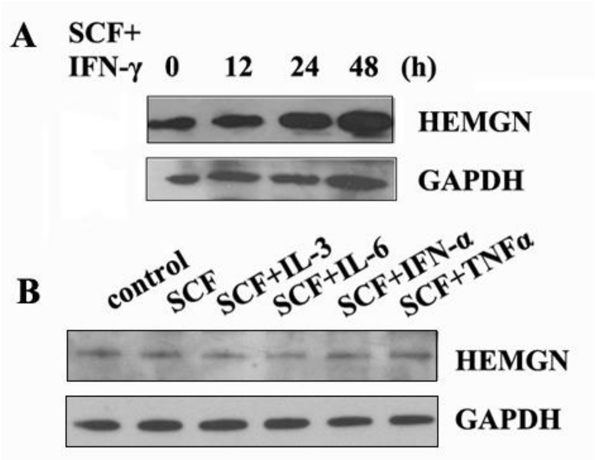

Figure S5. Sorted BM LSK cells were cultured in serum-free medium (SFEM) (Stem Cell Technologies, Cat#09650) supplemented with SCF (50ng/ml) plus IFN-γ (100ng/ml) (A) for the indicated time or with SCF (50ng/ml) plus the indicated inflammatory factors (IL-3: 20ng/ml, IL-6: 20ng/ml, IFN-α: 2×10^4U/ml, TNFα: 1μg/ml) for 48 hours (B). Freshly isolated LSK cells were used as control. The expression of HEMGN was analyzed by western blotting. GAPDH was used as internal control.

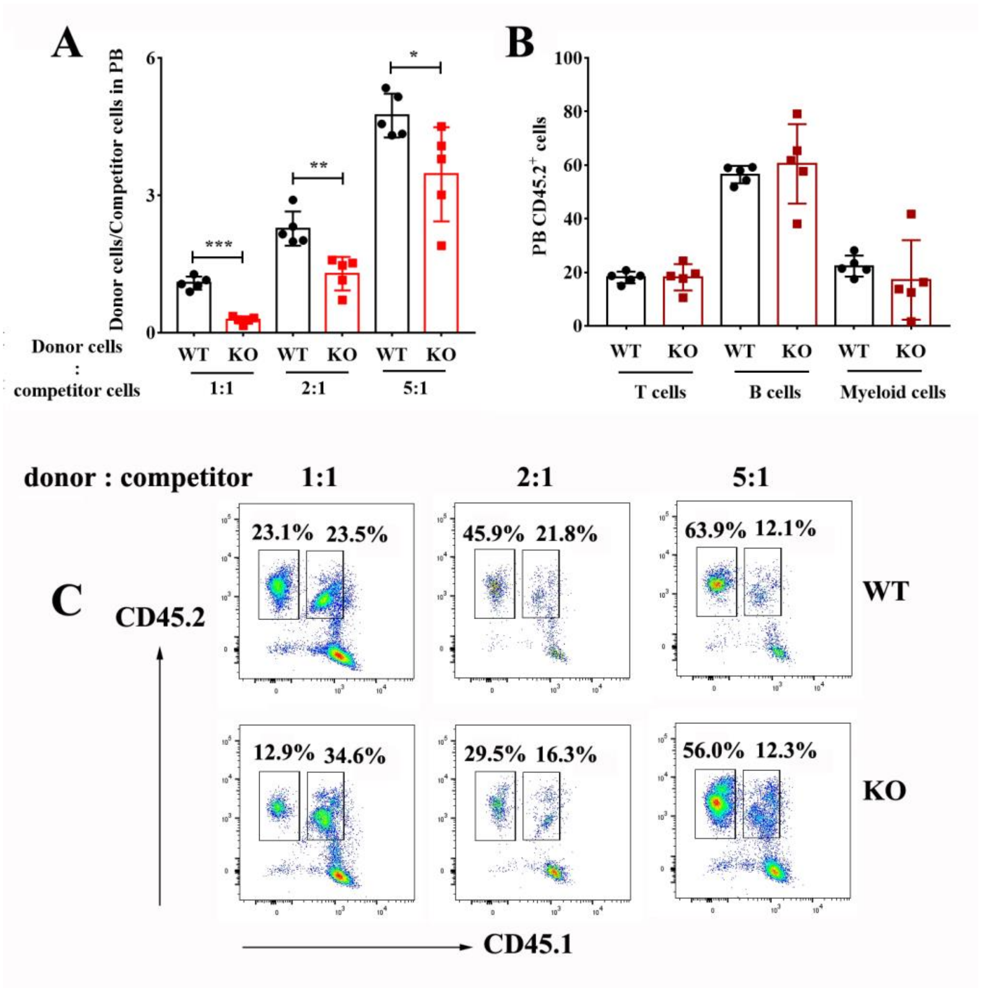

Figure S6. WT or *Hemgn*$^{-/-}$ mice BM mixed with competitor cells at the indicated ratio into the femoral BM cavity of lethally irradiated mice by intrafemoral transplantation. At 16w post-BMT, the ratio of donor cells to competitor cells in PB was analyzed (A). Ttrilineage differentiation in PB of recipients receiving donors and competitor cells at the ratio of 5:1 was analyzed (B). (C) Representative flow cytometric plots of PB at 16w after transplantation. n=5 mice per group. Data are the pool of two independent experiments. Error bars indicate SD.**P* < 0.05, ***P* < 0.01, ****P* < 0.001.

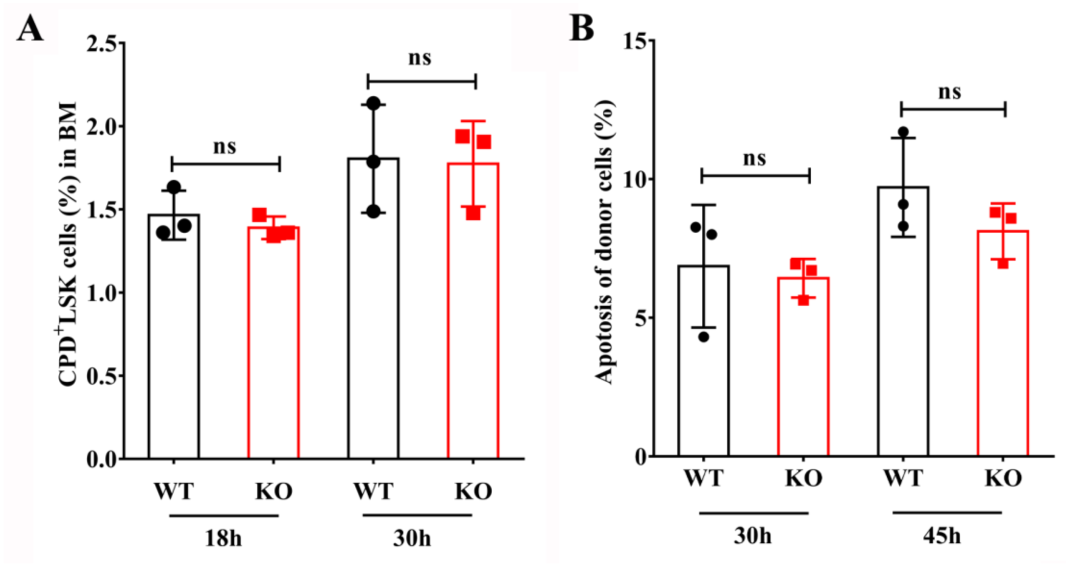

Figure S7. The homing efficiency and apoptosis of *Hemgn*$^{-/-}$ donor cells were comparable to WT donor cells in non-irradiated recipients. (A) CPD-labeled WT BM cells and *Hemgn*$^{-/-}$ BM cells (2×10^7) were transplanted into NOD/SCID mice without any conditioning. The percentage and apoptosis (B) of CPD$^+$ cells in BM were analyzed by flow cytometry at 30h post-transplantation. n=3 mice per group. Data are representative of 2 independent studies. Error bars indicate SD. *$P < 0.05$.

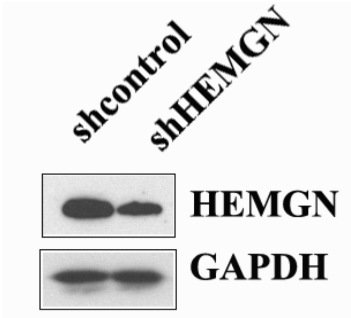

Figure S8. Validation of HEMGN knockdown in K562 cells.

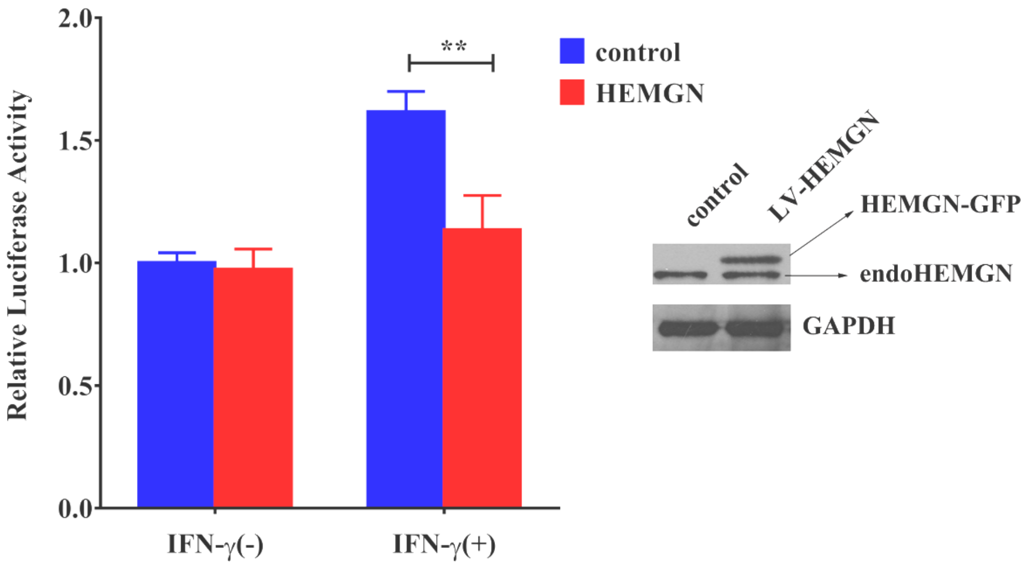

Figure S9. Control K562 cells or HEMGN lentivirus (LV-HEMGN)-infected K562 cells were transfected with GAS-reporter vector and then treated with IFN-γ (100ng/mL). The Luciferase activity was measured 12h later. The right panel shows the expression of endogenous HEMGN protein and HEMGN-GFP fusion protein in LV-HEMGN-infected K562 cells. Data are representative of three independent experiments. Error bars indicate SD. $*P < 0.05$, $**P < 0.01$.

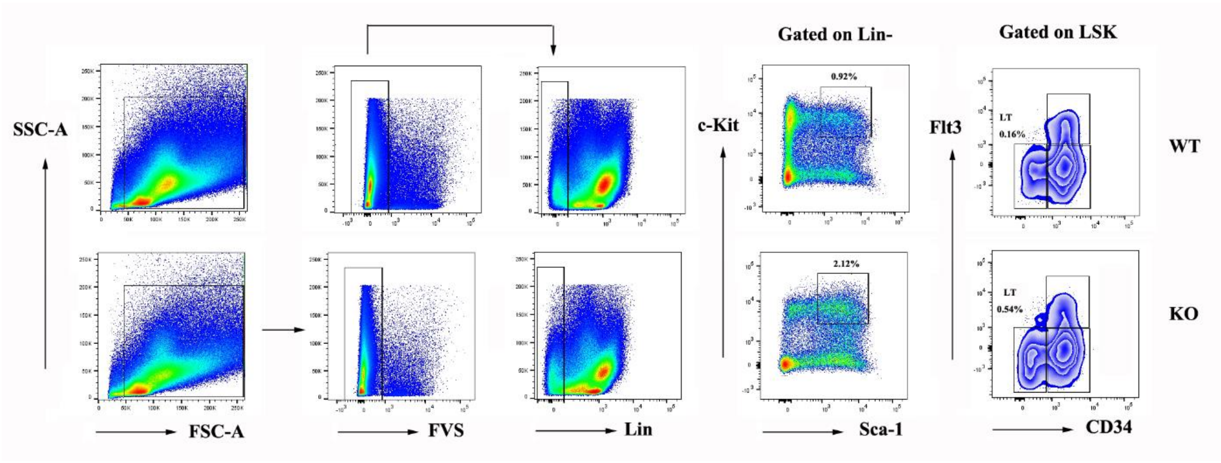

Figure S10. Gating strategy for BM HSPCs analysis of WT or *Hemgn*$^{-/-}$ mice injected with 5×10^6 CFU *Listeria monocytogenes* for 24h.

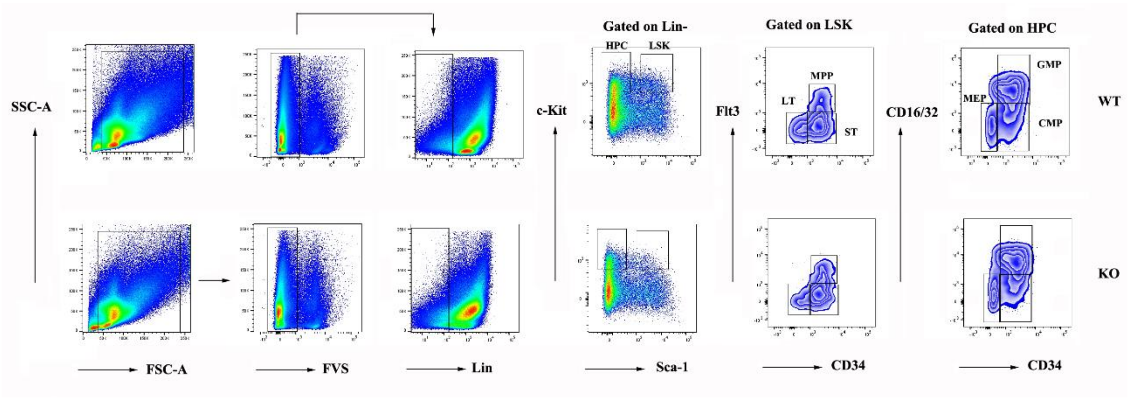

Figure S11. Gating strategy for BM HSPCs and committed progenitors analysis of 28-month-old mice.

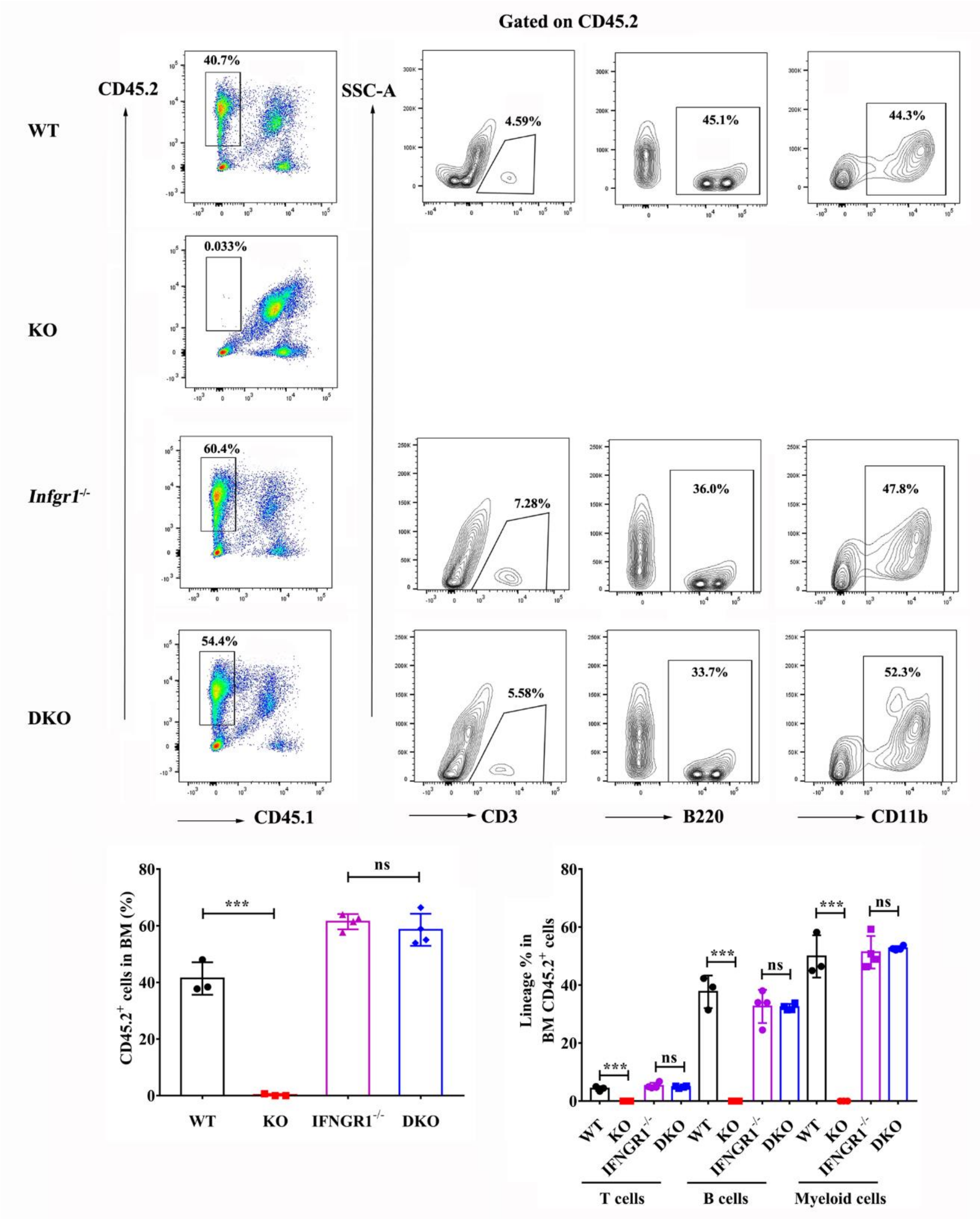

Figure S12. BM cells from WT (n=3 mice), *Hemgn*$^{-/-}$(KO) (n=3 mice), *Ifngr*1$^{-/-}$ (n=4 mice) or *Hemgn*$^{-/-}$*Ifngr*1$^{-/-}$(DKO) (n=4 mice) (5×10^6) were transplanted into irradiated recipients (CD45.1) in the presence of competitor cell (1×10^6). At 16w post-BMT, the chimerism and trilineage differentiation in BM were analyzed. Data are presented as mean ± SD. **P* < 0.05, ****P* < 0.001.

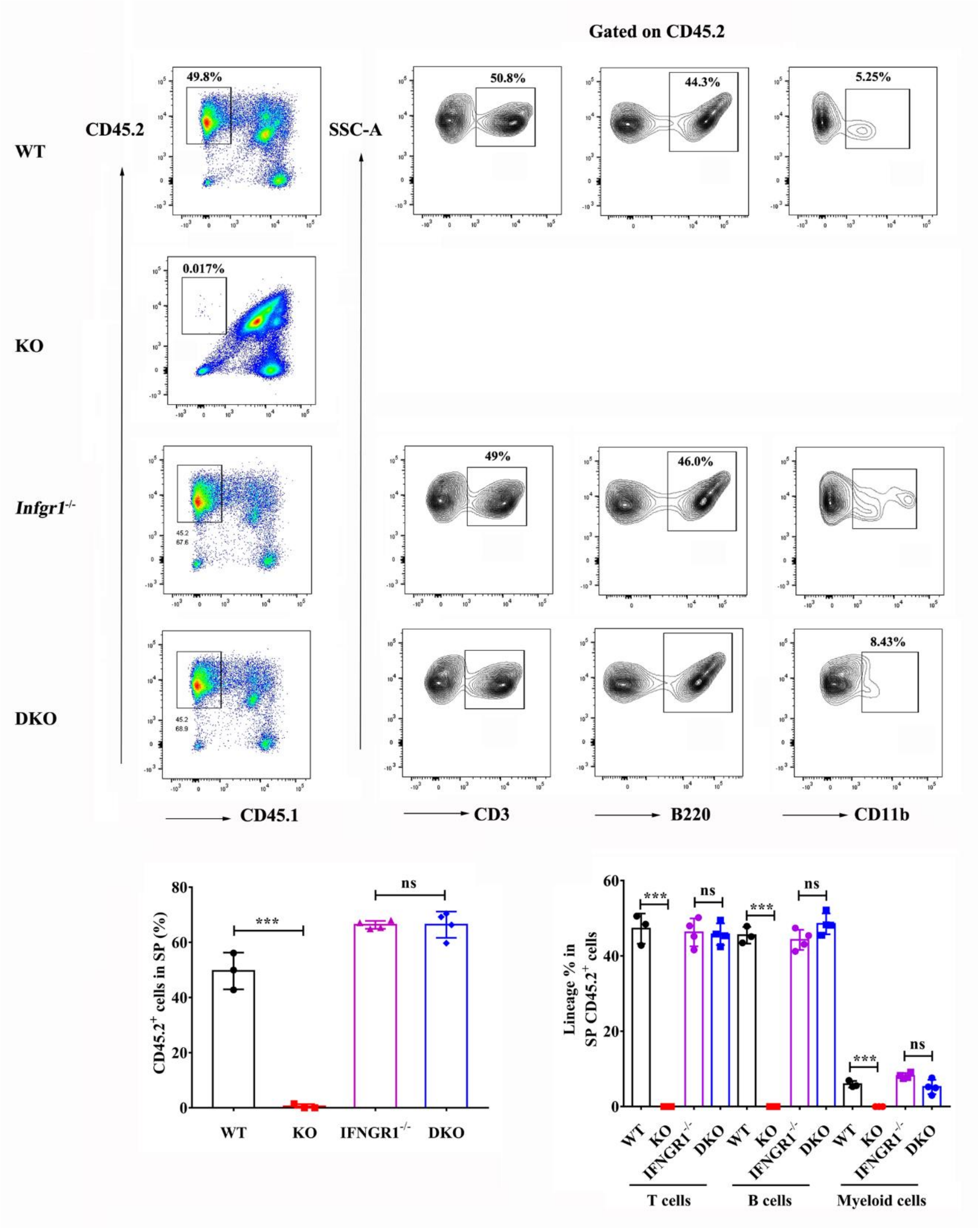

Figure S13. BM cells from WT (n=3 mice), *Hemgn*$^{-/-}$(KO) (n=3 mice), *Ifngr*1$^{-/-}$ (n=4 mice) or *Hemgn*$^{-/-}$*Ifngr*1$^{-/-}$(DKO) (n=4 mice) (5×10^{6}) were transplanted into irradiated recipients (CD45.1) in the presence of competitor cell (1×10^{6}). At 16w post-BMT, the chimerism and trilineage differentiation in SP were analyzed. Data are presented as mean ± SD. *P < 0.05, ***P < 0.001.

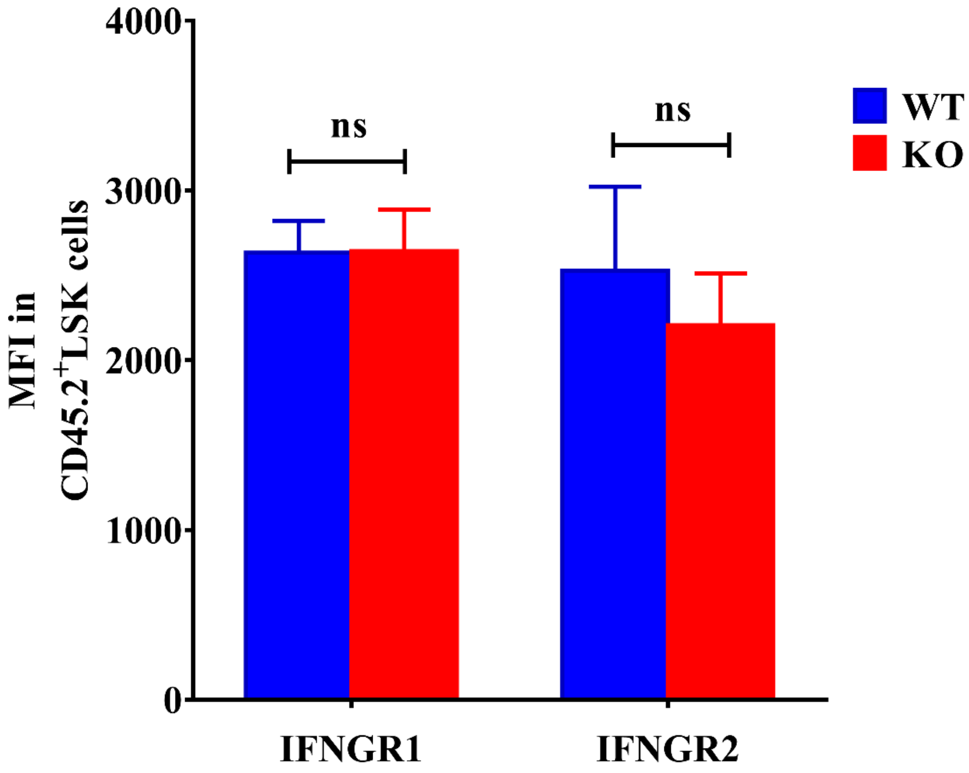

Figure S14. Analysis of IFNGR1 and IFNGR2 expression level in donor LSK cells from primary recipients BM by flow cytometry at 12h after BMT (n=4 mice per group). Data are the pool of two independent experiments. Error bars indicate SD. *P < 0.05.
