# Supplementary material for: Hemgn Protects Hematopoietic Stem and Progenitor Cells Against Transplantation Stress Through Negatively Regulating IFN‐γ Signaling
Source: Adv Sci (Weinh). 2021 Dec 19;9(5):2103838. doi: 10.1002/advs.202103838 (PMC8844507; doi:10.1002/advs.202103838)
Supplement: Supplementary file 3 — Supplemental Table 2 [file ADVS-9-2103838-s002.pdf]

Supplementary Table S1　Antibodies used in this study

Fluorescen	Antibody	Clone	Company	Cat.#
eFluor450	CD11b	M1/70	eBioscience	48-0112-82
BV500	FVS510	-	BD	564406
FITC	CD34	RAM34	eBioscience	11-0341-85
FITC	CD45.1	A20	Biolegend	110706
FITC	CD45.2	104	Biolegend	109806
FITC	Streptavidin	-	eBioscience	11-4317-87
PerCP	B22	RA3-6B2	Biolegend	103236
PE	CD45.1	A20	Biolegend	110708
PE	CD45.2	104	Biolegend	109808
PE	cKit（CD117）	2B8	eBioscience	12-1171-82
PE-CF594	CD135（Flt3）	A2F10.1	BD	562537
BV605	Sca-1	D7	BD	563288
BV605	CD45.1	A20	Biolegend	110738
PE-Cy7	Ki-67	B56	BD	561283
PE-Cy7	Sca-1	D7	eBioscience	25-5981-81
PE-Cy7	CD150	mShad150	eBioscience	25-1502-82
PE-Cy7	B220	RA3-6B2	eBioscience	25-0452-82
APC	cKit（CD117）	ACK2	eBioscience	17-1172-83
APC	CD3	145-2C11	eBioscience	17-0031-83
APC	CD45.2	104	Biolegend	109814
APC	Annexin V	-	KeyGEN	KGA1022
APC-eFlu	Streptavidin	-	eBioscience	47-4317-82
Biotin	CD11b	M1/70	eBioscience	13-0112-85
Biotin	Gr-1	RB6-8C5	eBioscience	13-5931-82
Biotin	B220	RA3-6B2	eBioscience	13-0452-82
Biotin	CD19	eBio1D3	eBioscience	13-0193-81
Biotin	TER119	TER-119	Biolegend	116204
Biotin	CD3	145-2C11	Biolegend	100304
Biotin	CD4	RM4-5	Biolegend	100508

Biotin	CD8	53-6.7	Biolegend	100704
PE	IFN γ R1	2E2	eBioscience	12-1191-82
PE	IFN γ R2	MOB-47	Santa Cruz	SC-12752
eFluor®67	CPD		eBioscience	65-0840-85
PerCP	CD127	A7R34	eBioscience	45-1271-82
	Anti-Stat1	D1K9Y	CST	14994
	Anti-Phospho- Stat1	Tyr701	CST	8826S
	Anti-Phospho- Stat1	Ser727	CST	9167S
	Anti-Jak1	D1T6W	CST	50996
	Anti-Phospho-Jak1	D7N4Z	CST	74129
	Anti-TCPTP	EPR6712	Abcam	Ab129070
	Anti-GAPDH		Proteintech	6004-1-IG
	Anti-Tubulin		Abclonal	AC021
	Anti-Lamin A/C	4C11	CST	4777S
